# 

**Published:** 1895-03

**Authors:** 


### THE BRISTOL

### Medico-Chirurgical Journal.

A JOURNAL OF THE MEDICAL SCIENCES FOR THE
WEST OF ENGLAND AND SOUTH WALES

*PUBLISHED UNDER THE AUSPICES OF*
*THE BRISTOL MEDICO-CHIRURGICAL SOCIETY.*

EDITOR:
R. SHINGLETON SMITH, M.D., B.Sc.
WITH WHOM ARE ASSOCIATED
J. MICHELL CLARKE, M.A., M.D.
F. R. CROSS, M.B. AND W. H. HARSANT.
ASSISTANT-EDITOR:
L. M. GRIFFITHS.
EDITORIAL SECRETARY:
B. M. H. ROGERS, B.A., M.D., B.Ch.

"Scire est nescire, nisi id me

Scire alius sciret."

*VOL. XIII.*

BRISTOL: J. W. ARROWSMITH;
LONDON: J. & A. CHURCHILL, 11 NEW BURLINGTON STREET.
1895.

R 31
B 8

J. W. Arrowsmith Publisher Bristol

3

### CONTENTS OF VOLUME XIII.

*Page*

Extra-Peritoneal Closure of Artificial Anus and Fæcal Fistula. By J. Greig Smith, M.A., M.B., C.M. Aberd., F.R.S.E. ... ... 1

Cystotomy for Bladder Tumours of Doubtful Malignancy. By J. Paul Bush, M.R.C.S. Eng. ... ... ... ... ... ... ... ... 8

The Administration of Nitrous Oxide as a Preliminary to Ether Anæsthesia. By Bertram M. H. Rogers, B.A., M.D., B.Ch. Oxon. ... ... ... ... ... ... ... ... ... ... ... ... ... ... 10

Some Clinical Notes on Membranous Enteritis. By C. Percival Crouch, M.B. Lond., F.R.C.S. Eng. ... ... ... ... ... ... 14

The Bacterioscopic Diagnosis of Diphtheria. By F. Wallis Stoddart, F.I.C., F.C.S. ... ... ... ... ... ... ... ... ... 18

Alcoholic Disease of the Heart. By Theodore Fisher, M.D. Lond. ... ... ... ... ... ... ... ... ... ... ... ... ... ... 81

The Paralysis of Osteo-Arthritis. By Henry Waldo, M.D. Aberd., M.R.C.P. Lond. ... ... ... ... ... ... ... ... ... ... ... 90

Buccal Glandular Tumours. By James Swain, M.D., M.S. Lond., F.R.C.S. Eng. ... ... ... ... ... ... ... ... ... ... ... ... 94

Sarcomatous Tumour of the Shoulder-Centre in the Cortex of the Right Hemisphere. By J. E. Shaw, M.B. Ed., and J. Paul Bush, M.R.C.S. Eng. ... ... ... ... ... ... ... ... ... ... 99

Four Cases of Blood-Poisoning in Brothers. By F. H. Edgeworth, B.A., M.B. Cantab., B.Sc. Lond. ... ... ... ... ... ... ... 107

Pneumococci in the Urine. By G. Munro Smith, M.R.C.S. Eng., L.R.C.P. Lond. ... ... ... ... ... ... ... ... ... ... ... 115

The Progress of Surgery in the last Twenty-five Years. By Horatio Percy Symonds ... ... ... ... ... ... ... ... ... ... ... 161

*Page*

On the Localisation of the Foramina at the Base of the Skull. By Edward Fawcett, M.B., C.M. Ed. ... ... ... ... ... ... 173

On the Diagnosis of Cancer and of Simple Dilatation of the Stomach. By J. Michell Clarke, M.A., M.D. Cantab., M.R.C.P. Lond. 181

The Administration of Ether. By John Freeman, F.R.C.S. Ed. ... 191

School Surgery. By Arthur W. Prichard, M.R.C.S. Eng. ... ... 241

Two Cases of Fibroid Degeneration of the Myocardium. By Theodore Fisher, M.D. Lond. ... ... ... ... .. ... ... 258

Notes on Egypt as a Health-Resort. By P. Watson Williams, M.D. Lond. ... ... ... ... ... ... ... ... ... ... ... ... 263

Progress of the Medical Sciences ... ... ... 24, 117, 196, 273

Reviews of Books ... ... ... ... ... ... ... 37, 141, 214, 292

Notes on Preparations for the Sick ... ... ... 65, 149, 231, 309

Meetings of Societies ... ... ... ... ... ... ... 68, 151, 312

The Library of the Bristol Medico-Chirurgical Society ... ... ... ... ... ... ... ... ... 73, 153, 234, 316

Obituary ... ... ... ... ... .. ... ... ... ... ... ... ... 76

Presentation to Dr. J. G. Swayne ... ... ... ... ... ... ... 78

Scraps ... ... ... ... ... ... ... ... ... ... 79, 159, 239, 323

Local Medical Notes ... ... ... ... ... ... ... 158, 237, 320

The William F. Jenks Memorial Prize ... ... ... ... ... ... 237

### The Library of the Bristol Medico-Chirurgical Society.

*The following donations have been received since the publication of the list in December:*

*February 28th*, 1895.

| | |
|---|---|
| Sir Thomas Dyke Acland, Bart. (1) ... ... ... | 1 volume. |
| J. Michell Clarke, M.D. ... ... ... ... ... ... | 23 volumes. |
| Obstetrical Society of London (2) ... ... ... ... | 2 „ |
| The Surgeon-General, United States Army (3) ... | 1 volume. |
| The Editors of the Pathological Report of University College, London (4) ... ... ... ... ... | 1 „ |
| J. Wright & Co. (5) ... ... ... ... ... ... ... | 1 „ |

Unbound periodicals have been received from Dr. Shingleton Smith.

### FIFTEENTH LIST OF BOOKS.

The titles of books mentioned in previous lists are not repeated.

The figures in brackets refer to the figures after the names of the donors, and show by whom the volumes were presented. The books to which no such figures are attached have either been bought from the Library Fund or received through the *Journal*.

| | | |
|---|---|---|
| **Anderson, J.** ... | *Medical Nursing.* (Ed. by Ethel F. Lamport.) 2nd Ed. | 1895 |
| **Bentley and Rev. C. G. Griffinhoofe, A. J. M.** | *Wintering in Egypt* ... ... | 1894 |
| **Black, D. C.** ... | *Address to the Physiology Class in Anderson's College. Session* 1891—92 ... ... ... ... ... ... ... ... | 1891 |
| **Boxall, R.** ... ... | *The Public, the Profession, and Medical Charities* ... | 1894 |
| **Burdett, H. C.** ... | *Helps in Sickness and to Health* ... ... ... ... ... | 1894 |
| *Catalogue (Index) of Library of Surgeon-General's Office, U.S. Army* | Vol. XV. (3) | 1894 |
| **Cerna, D.** ... ... | *Notes on the Newer Remedies.* ... .. ... 2nd Ed. | 1895 |
| **Cooper, A.** ... ... | *Syphilis.* 2nd Ed. (Ed. by E. Cotterell) ... ... | 1895 |
| **Creighton, C.** ... | *A History of Epidemics in Britain.* Vol. II. ... ... | 1894 |
| **Crookshank, E. M.** | *The Prevention of Small-pox* ... ... ... ... ... ... | 1894 |
| **Eardley-Wilmot, R.** | *On the Natural Mineral Waters and Spa of Leamington* | 1890 |
| **Feachem, Mary**... | *Food for the Sick and Convalescent* ... ... ... ... | 1895 |

**Felkin, R. W.** ... *On the Geographical Distribution of Tropical Disease in Africa* ... 1895
**Fenwick, E. H.** ... *Urinary Surgery* ... 1894
**Fink, G. H.** ... *Methods of Operating for Cataract* ... 1894
**Fletcher, R.** ... *Anatomy and Art* ... 1895
**Frerichs, F. T.** ... *Atlas of Pathological Anatomy Illustrative of Diseases of the Liver.* (Tr. and Ed. by C. Murchison.) Two Parts ... 1861-62
**Frothingham, L.** .. *Laboratory Guide for the Bacteriologist* ... 1895

**Gowers, W. R.** ... *The Dynamics of Life* ... 1894
**Griffinhoofe, A. J. M. Bentley and Rev. C. G.** *Wintering in Egypt* ... 1894

**Hart, E.** ... *The Sick Poor in Workhouses—Reports, &c.* First Series ... 1895
**Hewitt, F. W.** ... *Anæsthetics and their Administration* ... 1893
**Hutchinson, J.** ... *A Descriptive Catalogue of the New Sydenham Society's Atlas of Diseases of the Skin.* Two Parts ... 1869-75

**Long, J. W.** ... *Syllabus of Gynecology* ... 1895

**Martin, B. R.** ... *Diphtheria and its Treatment.* ... 2nd Ed. 1895
**Möller, F. P.** ... *Cod-Liver Oil and Chemistry* ... 1895
**Morton, A. S.** ... *Refraction of the Eye.* ... 5th Ed. 1894

**Oliver, G.** ... *Pulse-Gauging* ... 1895
**Oliver, J.** ... *Abdominal Tumours and Abdominal Dropsy in Women* 1895
**Ónodi, A.** ... *The Anatomy of the Nasal Cavity* ... 1895

*Pathology, An Atlas of Illustrations of* (New Syd. Soc.). Fasc. I.—IX. 1877-94

**Schimmelbusch, C.** *The Aseptic Treatment of Wounds.* (Tr. by A. T. Rake) 1894
**Sheild, A. M.** .. *Diseases of the Ear* ... 1895
**Swanzy, H. R.** ... *Diseases of the Eye.* 5th Ed. (Ed. by L. Werner.) 1895
*Sydenham in the Oxford Museum, The Unveiling of* ... (1) 1894

**Tomlins, W. H.** ... *The Droitwich Brine Baths* ... 1895

**Waldo and D. Walsh, F. J.** *Bread, Bakehouses, and Bacteria* ... 1895
**Walsh, F. J. Waldo and D.** *Bread, Bakehouses, and Bacteria* ... 1895

---

### TRANSACTIONS, REPORTS, JOURNALS, &c.

American Journal of Obstetrics, The ... Vol. XXX. 1894

American Journal of the Medical Sciences, The ... Vol. CVIII. 1894

American Surgical Association, Transactions of the ... Vol. XII. 1894

Archives de Neurologie ... Tome XXVIII. 1894

Birmingham Medical Review, The ... Vol. XXXVI. 1894

Boston Medical and Surgical Journal, The ... Vol. CXXXI. 1894

Bristol and Clifton Directory ... (5) 1895

British Journal of Dermatology, The ... Vol. VI. 1894

British Medical Journal, The ... Vol. II. for 1894

Canadian Practitioner, The ... Vol. XIX. 1894

| | | |
|---|---|---|
| Cincinnati Lancet-Clinic, The | Vol. LXXII. | 1894 |
| Clinical Journal, The | Vol. IV. | 1894 |
| Directory (International) of Second-hand Booksellers, The | | 1894 |
| Dublin Journal of Medical Science, The | Vol. XCVIII. | 1894 |
| Gazette de Gynécologie | Tome IX. | 1894 |
| Gazette des Hôpitaux | | 1894 |
| Glasgow Medical Journal, The | Vol. XLII. | 1894 |
| Hazell's Annual | | 1895 |
| Hospitals— | | |
| Middlesex Hospital, The—Reports of the Medical and Surgical Registrars and Pathologist for 1893 | | 1894 |
| Saint Thomas's Hospital Reports | Vol. XXII. | 1894 |
| University College, London.—Pathological Report. | (4) Vol. IV. | 1894 |
| Westminster Hospital Reports, The | Vol. IX. | 1895 |
| Journal of Cutaneous and Genito-Urinary Diseases | Vol. XII. | [1894] |
| Journal of Laryngology, Rhinology, and Otology, The | Vol. VIII. | 1894 |
| Journal of Nervous and Mental Disease, The | Vol XIX. | 1894 |
| Lancet, The | Vol. II. for | 1894 |
| Mathews' Medical Quarterly | Vol. I. | 1894 |
| Medical Age, The | Vol. XII. | 1894 |
| Medical and Surgical Reporter, The | Vol. LXXI. | 1894 |
| Medical Chronicle, The | N.S. Vol. I. | 1894 |
| Medical Directory, The | | 1895 |
| Medical News, The | Vol. LXV. | 1894 |
| Medical Press and Circular, The | Vol. CIX. | 1894 |
| Medical Record | Vol. XLVI. | 1894 |
| Medical Society of London, Transactions of the | Vol. XVII. | 1894 |
| Medico-Chirurgical Transactions | Vol. LXXVII. | 1894 |
| Münchener Medicinische Wochenschrift | | 1894 |
| New York Medical Journal, The | Vol. LX. | 1894 |
| Obstetrical Society of London, Transactions of the. | (2) Vols. XXXIV., XXXV., 1893-94; Vol. XXXVI., Parts I.—III. | 1894 |
| Post-Graduate, The | Vol. IX. | [1894] |
| Practitioner, The | Vol. LIII. | 1894 |
| Progrès Médical, Le | Tome XX. | 1894 |
| Retrospect of Medicine, Braithwaite's | Vol. CX. | 1895 |
| Revue de Laryngologie, d'Otologie et de Rhinologie | Tome XIV. | 1894 |
| Revue générale d'Ophtalmologie | Tome XIII. | 1894 |
| Teratologia | Vol. I. | 1894 |
| Therapeutic Gazette, The | Vol. XVIII. | 1894 |
| Union médicale, L' | Tome LVIII. | 1894 |
| University Medical Magazine | Vol. VI. | 1894 |
| Victoria— | | |
| Nosological Index | | 1862-3 |
| Vital Statistics of the Colony of Victoria for 1872 | | 1874 |
| " " " 1874 | | 1875 |
| Whitaker's Almanack | | 1895 |
| Wiener klinische Wochenschrift | | 1894 |
| Zeitschrift für Krankenpflege | | 1894 |

### Bristol Medico-Chirurgical Society.

---

**President.**

A. J. HARRISON, M.B. Lond.

**President-Elect.**

ARTHUR W. PRICHARD.

**Honorary Secretary.**
J. PAUL BUSH.

**Committee.**

*Past Presidents.*
R. W. COE.
AUGUSTIN PRICHARD, M.D. Berlin.
J. G. SWAYNE, M.D. Lond.
E. LONG FOX, M.D. Oxon.
W. JOHNSTONE FYFFE, M.D., B.A. Dub.
W. H. SPENCER, M.D., M.A. Cantab.
F. POOLE LANSDOWN.
L. M. GRIFFITHS.
R. SHINGLETON SMITH, M.D., B.Sc. Lond.
NELSON C. DOBSON.
S. H. SWAYNE.
F. R. CROSS, M.B. Lond.
E. MARKHAM SKERRITT, M.D., B.S., B.A. Lond.
J. GREIG SMITH, M.B., C.M., M.A. Aberd., F.R.S.E.

J. MICHELL CLARKE, M.D., M.A. Cantab.
JOHN DACRE.
W. H. HARSANT.
A. E. AUST LAWRENCE, M.D., C.M. Aberd.
J. E. SHAW, M.B., C.M. Ed.
G. MUNRO SMITH.

Medical Practitioners wishing to join the Society are requested to communicate with the Honorary Secretary, J. PAUL BUSH, Vyvyan House, Clifton Park, Clifton, Bristol. The Annual Subscription is 21/-.

---

*Extracts from Journal By-laws.*

4.—The *Journal* shall be delivered free to Subscribers and also to Members of the Society whose subscriptions are not in arrear.

6.—The Annual Subscription to the *Journal* for those who pay before the 1st of July shall be 5/-, post free, or 6/- if paid after that date.

---

Communications intended for insertion in the *Journal* should be sent to the Editor, Dr. SHINGLETON SMITH, Deepholm, Clifton Park, Clifton, Bristol.

Books for Review and Exchange Journals should be sent to the Assistant-Editor, L. M. GRIFFITHS, 9 Gordon Road, Clifton, Bristol.

Communications referring to the delivery of the *Journal* should be sent to the Editorial Secretary, Dr. BERTRAM ROGERS, 11 York Place, Clifton, Bristol, to whom Subscriptions are to be paid *in advance*.

Cases for Binding Vols. I.–XII. are now ready, and may be obtained, price 7*d*. each (postage 2*d*. extra), upon application to J. W. ARROWSMITH, Quay Street, Bristol; or the *Journal* will be bound, including Case, for 1/3 per volume.

### Bristol Medico-Chirurgical Society.

### PAST PRESIDENTS.

1874—75. FREDERICK BRITTAN, M.D., B.A. Dub.

1875—76. ROBERT WILLIAM COE.

1876—77. SAMUEL MARTYN, M.D. Ed.

1877—78. AUGUSTIN PRICHARD, M.D. Berlin.

1878—79. HENRY EDWARD FRIPP, M.D. Würzburg.

1879—80. WILLIAM MICHELL CLARKE.

1880—81. JOSEPH GRIFFITHS SWAYNE, M.D. Lond.

1881—82. EDWARD LONG FOX, M.D. Oxon.

1882—83. JAMES GEORGE DAVEY, M.D. St. And.

1883—84. WILLIAM JOHNSTONE FYFFE, M.D., B.A. Dub.

1884—85. GEORGE FORSTER BURDER, M.D. Aberd.

1885—86. WILLIAM HENRY SPENCER, M.D., M.A. Cantab.

1886—87. FRANCIS POOLE LANSDOWN.

1887—88. LEMUEL MATTHEWS GRIFFITHS.

1888—89. ROBERT SHINGLETON SMITH, M.D., B.Sc. Lond.

1889—90. NELSON CONGREVE DOBSON.

1890—91. SAMUEL HENRY SWAYNE.

1891—92. FRANCIS RICHARDSON CROSS, M.B. Lond.

1892—93. EDWARD MARKHAM SKERRITT, M.D., B.S., B.A. Lond.

1893—94. JAMES GREIG SMITH, M.B., C.M., M.A. Aberd., F.R.S.E.

1895.

### LIST OF SUBSCRIBERS

TO THE

### Bristol Medico-Chirurgical Journal.

*Those marked * are Members of the Society.*

| | |
|---|---|
| Ackland, J. McKno | 24 Southernhay, Exeter. |
| *Ackland, W. R. | 5 Rodney Place, Clifton. |
| Adams, George | 11 Westbourne Place, Clifton. |
| *Adams, John Barfield | 47 Redand Road, Bristol. |
| Adye, W. J. A. | Church House, Bradford, Wilts. |
| Aldridge, Charles, M.D., C.M. Aberd. | Plympton. |
| Aldous, George F. | Charlton House, Mannamead, Plymouth. |
| Alexander, James, M.D., M.Ch., B.A. Dub. | Bishop's Place, Paignton. |
| *Alford, George Ernest | 5 Royal Crescent, Weston-super-Mare. |
| Anstie, Thomas B. | 21 Northgate Street, Devizes. |
| *Atchley, Geooge F., M.B. Lond. | Tyndall's Park, Bristol. |
| Avarne, A. B. | Blaenavon, Monmouthshire. |
| *Aveline, H. T. S. | Asylum, Stapleton, Gloucestershire. |
| | |
| Ballantyne, J. W., M.D., C.M. Ed. | 24 Melville Street, Edinburgh. |
| Bannatyne, Gilbert A., M.D., C.M. Glasg. | 1 Paragon, Bath. |
| *Barclay, W. M. | Queen's Road, Clifton. |
| Baretti, T. G. L'Enardi | 49 Royal York Crescent, Clifton. |
| *Baron, Barclay J., M.B., C.M. Ed. | 16 White Ladies Road, Clifton. |
| Barr, J. Coleman | 2 Cranmore Cottages, Aldershot. |
| *Basset, Walter | 24 Stow Hill, Newport, Mon. |
| *Bateman, F. J. B. | Alburgh House, Wells, Somersetshire. |
| Batten, Rayner W., M.D. Lond. | 1 Brunswick Square, Gloucester. |
| Batten, W. Smith | Curzon House, Calne. |
| *Beddoe, John, M.D. Ed., B.A. Lond., F.R.S. | The Chantry, Bradford-on-Avon. |
| Benham, Harry A., M.D., C.M. Aberd. | Asylum, Stapleton, Gloucestershire. |
| Bennett, W. S. | Escot, Penzance. |
| *Benson, A. H. | Wrington, Somersetshire. |
| Berry, F. C., M.D., B.Ch., B.A. Dub. | Burnham, Somersetshire. |
| *Berry, James, M.B., B.S. Lond. | 60 Welbeck Street, London, W. |
| Bevan, W. L. P., M.D., C.M. Ed. | Alton, Hampshire. |
| *Blacker, A. E. | Richmond Hill Avenue, Clifton. |
| *Blacker, E. | 60 St. Mary's Road, Southampton. |
| *Blagg, Arthur F. | 6 West Mall, Clifton. |
| Blomfield, Arthur G., M.D. Aberd. | 34 West Southernhay, Exeter. |
| *Board, Edmund C. | 11 Caledonia Place, Clifton. |
| *Boyce, James G. | The Chipping, Wotton-under-Edge. |
| Boys, Arthur Henry | Chequer Street, St. Alban's. |

### LIST OF SUBSCRIBERS.

| | |
|---|---|
| Brabazon, A. B., M.D. Aberd. ... ... ... ... | 12 Darlington Street, Bath. |
| Bramonti, Sebastiano... ... ... ... ... ... ... | 27 Via Balbi, Genoa, Italy. |
| *Brasher, Charles W. J. ... ... ... ... | 144 Cheltenham Road, Bristol. |
| Bright, J. A. ... ... ... ... ... ... ... ... ... ... | Glastonbury. |
| *Broom, John, M.D., Ch.D., Obst.D. Brussels | St. Paul's Road, Clifton. |
| Brown, G. Arthur ... ... ... ... ... ... ... | Tredegar. |
| Brown, William, M.D., C.M. Glasg. ... ... | Fishponds, Gloucestershire. |
| Browne, J. Walton, M.D., B.A. Roy. Univ. I. | 10 College Square North, Belfast. |
| Burroughs, E. F. H. ... ... ... ... ... ... ... | 3 Elgin Park, Bristol. |
| *Bush, J. Paul ... ... ... ... ... ... ... ... ... | Clifton Park, Clifton. |
| Cameron, John, M.B., C.M. Glasg. ... ... | Ashley Hill, Bristol. |
| Campbell, George... ... ... ... ... ... ... ... | Chilton Polden. |
| *Carr, Alexander ... ... ... ... ... ... ... ... | 150 Wells Road, Bristol. |
| *Carter, T. M. ... ... ... ... ... ... ... ... ... | 13 Dowry Square, Clifton. |
| *Carter, W. F.... ... ... ... ... ... ... ... ... | 48 Coronation Road, Bristol. |
| Cave, Edward J., M.D. Lond. ... ... ... ... | Crewkerne. |
| Cheves, Alexander B., M.D., M.A. Aberd. ... | Millbrook, Devonshire. |
| *Christie, W. L., M.D. Otago, N.Z. ... ... | Children's Hospital, Bristol. |
| Church, Basil... ... ... ... ... ... ... ... ... | Minchinhampton, Gloucestershire. |
| *Clarke, John Michell, M.D., M.A. Cantab. | 28 Pembroke Road, Clifton. |
| Clay, Challoner ... ... ... ... ... ... ... ... | Manor House, Fovant. |
| Clay, R. Hogarth, M.D. Ed. ... ... ... ... | 4 Windsor Villas, Plymouth. |
| Coathupe, Edwin W. ... ... ... ... ... ... ... | 7 Clifton Park, Clifton. |
| Cobbold, C. S. W., M.D. Würzburg ... ... | Bailbrook House, Bath. |
| *Coe, R. W. ... ... ... ... ... ... ... ... ... ... | 7 Pembroke Road, Clifton. |
| Coker, T. V. ... ... ... ... ... ... ... ... ... ... | 2 Chesterfield Place, Clifton. |
| Cole, Thomas, M.D. Lond. ... ... ... ... | 18 Circus, Bath. |
| *Collins, E. Tenison ... ... ... ... ... ... ... | 12 Windsor Place, Cardiff. |
| *Collins, H. W. ... ... ... ... ... ... ... ... ... | Wrington, Somersetshire. |
| Colman, Henry ... ... ... ... ... ... ... ... | Pewsey, Wiltshire. |
| Colmer, Ptolemy S. H., M.D. Dur. ... ... | South Street House, Yeovil. |
| *Cook, Henri, M.B., C.M. Aberd. ... ... ... | 45 Stackpool Road, Bristol. |
| Cooke, A. S. ... ... ... ... ... ... ... ... ... ... | Badbrook House, Stroud. |
| *Corbould, George G. ... ... ... ... ... ... | 22 Berkeley Square, Bristol. |
| Cossham, W. R., M.D., C.M. Aberd.... ... ... | Cirencester. |
| Cotton, William, M.B., C.M., M.A. Ed. ... | 227 Gloucester Road, Bristol. |
| Cousins, J. Ward, M.D. Lond. ... ... ... | Kent Road, Southsea. |
| Cowan, F. S. ... ... ... ... ... ... ... ... ... ... | 27 Queen Square, Bath. |
| Craddock, Samuel ... ... ... ... ... ... ... | South Lyncombe, Bath. |
| Crespi, Alfred J. H. ... ... ... ... ... ... | Poole Road, Wimborne. |
| *Cross, F. Richardson, M.B. Lond. ... ... | Worcester House, Clifton. |
| *Crossman, Edward, M.D. Dur. ... ... ... | Hambrook, Gloucestershire. |
| Crouch, C. Percival, M.B. Lond. ... ... ... | 3 Princes Buildings, Weston-super-Mare. |
| Culross, James, M.B., C.M., M.A. Glasg. ... | 66 Queen Street, Newton Abbot. |
| Cunningham, C. L. ... ... ... ... ... ... | Chudleigh. |
| *Dacre, John ... ... ... ... ... ... ... ... ... | 14 Eaton Crescent, Clifton. |
| Daniel, T. P. ... ... ... ... ... ... ... ... ... ... | Beaminster, Dorsetshire. |
| *Davies, D. S., M.D. Lond. ... ... ... ... | 60 Oakfield Road, Clifton. |
| Davies, Ebenezer... ... ... ... ... ... ... ... | Brunswick House, Swansea. |
| Davies, H. N. ... ... ... ... ... ... ... ... ... | Porth, Glamorganshire. |
| Davis, Robert ... ... ... ... ... ... ... ... ... | Oakleigh, Epsom. |
| Davis, Theodore, M.D. Lond. ... ... ... | Beachcroft, Clevedon. |
| Davy, Henry, M.D. Lond.... ... ... ... ... ... | Southernhay House, Exeter. |
| Day, Percy Howard ... ... ... ... ... ... ... | Stalmine, Poulton-le-Fylde. |
| Deas, P. Maury, M.B., M.S. Lond. ... ... | Wonford House, Exeter. |
| De'Ath, G. H. ... ... ... ... ... ... ... ... ... | Buckingham. |
| Dening, Edwin ... ... ... ... ... ... ... ... | Manor House, Stow-on-the-Wold. |
| *Devis, Harry F. ... ... ... ... ... ... ... ... | Wells Road, Bristol. |

### LIST OF SUBSCRIBERS.

| | |
|---|---|
| Dickie, James Steel, M.D., C.M. Aberd. | Christchurch Road, Bournemouth. |
| *Dobson, Nelson C. | 27 Victoria Square, Clifton. |
| Domville, Edward J. | 44 Southernhay, Exeter. |
| *Dowson, W., M.D., B.C., M.A. Cantab. | Elgin Park, Bristol. |
| Doyne, Robert W. | St. Giles, Oxford. |
| Duffett, E. D. | 5 London Street, Swindon, Wiltshire. |
| Dunbar, Eliza L. Walker, M.D. Zürich | 9 Oakfield Road, Clifton. |
| *Duncan, William | Westbury-on-Trym. |
| | |
| Eadon, W. F. Bailey | Hambrook, Gloucestershire. |
| *Eager, Reginald, M.D. Lond. | Northwoods, Winterbourne. |
| Eddowes, Charles | Maddington, Devizes. |
| *Edgeworth, F. H., M.B., B.C., B.A. Cantab., B.Sc. Lond. | 1 Richmond Hill, Clifton. |
| Edis, Thomas | 66 Barton Street, Gloucester. |
| Edwards, W. T., M.D. Lond. | 129 Queen Street, Cardiff. |
| Elder, William | 7 Trelawney Road, Bristol. |
| *Elliott, Christopher, M.D., B.A. Dub. | 88 Pembroke Road, Clifton. |
| Ellis, W. McD., M.D. Brussels | 7 Alexander Buildings, Bath. |
| Elsworth, R. C., M.B., C.M. Ed. | 203 St. Helen's Road, Swansea. |
| Embleton, Dennis C. | St. Michael's Road, Bournemouth. |
| *Etches, W. R. | Macclesfield. |
| Evans, J. Fenton, M.B., C.M. Ed. | 6 Whitehall Yard, London, S.W. |
| *Ewens, John | The Elms, Cotham Hill, Bristol |
| | |
| Fairbanks, W., M.D., C.M. Ed. | Wells, Somersetshire. |
| *Fendick, R. George | St. Paul's Road, Clifton. |
| Fergusson, W. Balfour, M.D., C.M. Ab. | Hazelbury House, Painswick. |
| Finch, Thomas, M.B., B.A. Cantab. | Watcombe View, Babbacombe. |
| *Firth, J. L., M.D., B.S. Lond. | General Hospital, Bristol. |
| *Fisher, Theodore, M.D. Lond. | 25 Pembroke Road, Clifton. |
| *Flemming, C. E. S. | Freshford. |
| Ford, Joseph | Wedmore. |
| Forty, D. H. | Wotton-under-Edge. |
| Fowler, O. H. | Cirencester. |
| Fox, Arthur E. W., M.B., C.M. Ed. | 16 Gay Street, Bath. |
| *Fox, Bonville B., M.D., M.A. Oxon. | Brislington. |
| *Fox, E. Long, M.D. Oxon. | Church House, Clifton |
| Fox, John Alfred | Morrab Road, Penzance. |
| Fox, T. Colcott, M.B. Lond., B.A. Cantab. | 14 Harley Street, London, W. |
| Fraser, Græme B. | Beach Road, Weston-super-Mare. |
| Freeman, Henry W. | 24 Circus, Bath. |
| Fry, J. Blount | Ashley Lodge, Esher. |
| *Fyffe, W. Johnstone, M.D., B.A. Dub. | 2 Rodney Place, Clifton. |
| | |
| Gamble, C. H. | Barnstaple. |
| Garner, John E., M.D., C.M. Aberd. | 6 Wenckley Square, Preston, Lancashire. |
| George, R. Julyan, M.B., C.M. Ed. | Port Isaac, Cornwall. |
| *Gibbs, Alfred N. Godby | 52 Whiteladies Road, Clifton. |
| Gill, John, M.D. Brussels | 75 Pembroke Road, Clifton. |
| Gloag, George A. | 7 College Green, Bristol. |
| Goodfellow, W. R. | Roche, Cornwall. |
| Goodridge, Henry F. A., M.D. Lond. | 10 Brock Street, Bath. |
| Goodwyn, A. H. | Droxford, Hampshire. |
| Gordon, Colin, M.B., C.M. Edin. | 15 Redland Grove, Bristol. |
| Goss, T. Biddulph | 1 Circus, Bath. |
| Grace, Alfred | Chipping Sodbury. |

### LIST OF SUBSCRIBERS.

| | | |
|---|---|---|
| Grace, Edward Mills | ... | Thornbury, Gloucestershire. |
| Grace, Henry | ... | Kingswood, Bristol |
| Grace, W. G. | ... | 15 Victoria Square, Clifton. |
| Granville, J. Mortimer, M.D. St. And. | ... | 14 Hanover Square, London, W. |
| Gratte, C. Brooke | ... | 3 Lansdowne Place, Newport, Mon. |
| *Gray, A. Murray | ... | 17 Market Place, Devizes. |
| Greenway, A. G., M.D. R.U.I. | ... | Llandridod Wells, Radnorshire. |
| Grey, T. C. | ... | The Shrubbery, Weston-super-Mare. |
| Griffiths, C. A. | ... | General Hospital, Swansea. |
| *Griffiths, J. S. | ... | 25 Redland Park, Bristol. |
| *Griffiths, L. M. | ... | 9 Gordon Road, Clifton. |
| Griffiths, P. Rhys, M.B., B.S. Lond. | ... | 71 Newport Road, Cardiff. |
| Grote, G. W., M.B. Toronto | ... | 36 Duffern Road, Upper Tooting, London, S.W. |
| Groves, J., M.B., B.A. Lond. | ... | Carisbrooke. |
| | | |
| Hadwen, W. R. | ... | Highbridge, Somersetshire. |
| *Hailes, Clements, D. G., M.D., C.M. Ed. | ... | 9 King's Parade, Clifton. |
| *Haines, A. H. | ... | 8 Cotham Road, Bristol. |
| Hale, Edmund T. | ... | The Grange, Chew Magna. |
| Halpin, W. C. | ... | Parkend. |
| Hamlen-Williams, T. R. | ... | Verlands, Cowbridge. |
| Handfield-Jones, Montagu, M.D. Lond. | ... | 35 Cavendish Square, London, W. |
| Hardwick, A., M.B. Dur. | ... | Newquay, Cornwall. |
| Harper, Joseph | ... | Bear Street, Barnstaple. |
| *Harrison, A. J., M.B. Lond. | ... | Guthrie Road, Clifton. |
| Harrison, Charles | ... | Keynsham, Somersetshire. |
| Harsant, J. G., M.D., B.S. Lond. | ... | Exeter Road, Bournemouth. |
| *Harsant, W. H. | ... | 16 Pembroke Road, Clifton. |
| *Harvey, Alfred, M.B. Lond. | ... | Westbury-on-Trym. |
| Hawkins-Ambler, G. A. | ... | 162 Upper Parliament Street, Liverpool. |
| Hawkins, Cæsar F. | ... | North Petherton. |
| Hayes, H. W. McCaulty | ... | 2 Prince's Square, London, W. |
| Hayes, W. A. | ... | Elm Grove, Calne. |
| Hayman, Alfred G. | ... | 1 Pembroke Road, Clifton. |
| *Hayman, C. A. | ... | 17 Victoria Square, Clifton. |
| Heane, W. C. | ... | Cinderford. |
| *Heaven, John C. | ... | 17 Whiteladies Road, Bristol. |
| Hemming, J. Hughes | ... | Kimbolton, St. Neot's. |
| *Herapath, C. K. C. | ... | Cotham Park, Bristol. |
| *Hetling, H. E. | ... | Kingsley Road, Bristol. |
| Hill, Hedley | ... | 1 Redcliff Hill, Bristol. |
| Hill, Thomas | ... | 138 Cromwell Road, Bristol. |
| *Hill, Walter J. | ... | Royal Infirmary, Bristol. |
| Hills, Frederic F. | ... | 146 Stapleton Road, Bristol. |
| Hoblyn, Francis Parker | ... | 10 Bladud Buildings, Bath. |
| *Hoffman, A. W. W. | ... | 30 The Parade, Leamington. |
| Horsfall, John, M.A. Oxon. | ... | Richmond Hill, Bournemouth. |
| Hospital, Bristol General (*Sec.*, W. Thwaites). | | |
| Hospital, Cincinnati, U.S.A. | | |
| Hospital, Taunton and Somerset (*House-Surgeon*, J. G. Bain). | | |
| Howells, William, M.B., C.M. Glasg. | ... | Church House, Talgarth, Breconshire. |
| Howse, William | ... | 8 London Street, Swindon, Wiltshire. |
| Howsin, E. Arthur, M.D. St. And. | ... | Stroud. |
| Hudson, F. Horace | ... | Sturminster Newton. |
| Hudson, H. R. | ... | 182 Commercial Road, Newport, Mon. |
| Huggard, W. R., M.D., M.Ch., M.A. Roy. Univ. I. | | Davos-Platz, Switzerland. |
| Humphreys, Henry, M.B., B.A. Cantab. | ... | St. Mary's Church Road, Torquay. |
| Hunt A. D. | ... | Millbrook House, Chagford. |
| Hyatt, J. T. | ... | Shepton Mallet. |

### LIST OF SUBSCRIBERS.

| | |
|---|---|
| *Imlay, G. A., M.B., C.M. Aberd. ... ... ... | 1 Cotham Park, Bristol. |
| Institution, Liverpool Medical (*Resident Librarian*, William Jones). | |
| Ives, William R. Y. ... ... ... ... ... ... | Portswood Road, Southampton. |
| | |
| Jackson, Mark, M.D. Roy. Univ. I.... ... ... | Bear Street, Barnstaple. |
| Jacobson, W. H. A., M.B., M.C., M.A. Oxon. | 66 Great Cumberland Place, London, W. |
| James, J. Bowen ... ... ... ... ... ... ... | Woodlands, Aberaman, Aberdare. |
| Jamison, A., M.B., B.Ch., M.A. Roy. Univ. I. | Belfast. |
| Jessop, Charles Moore ... ... ... ... ... | Clare Lodge, Redhill. |
| Johnson, G. H. ... ... ... ... ... ... ... | Teignmouth. |
| *Johnston, D. G., M.B., C.M. Glasg. ... ... | 92 Hampton Road, Bristol. |
| Jones, A. Orlando, M.D., C.M. Aberd. ... | Harrogate. |
| Jones, Evan ... ... ... ... ... ... ... ... | Ty-mawr, Aberdare. |
| Jones, H. Macnaughton, M.D., M.Ch. Roy. Univ. I. ... ... ... ... ... ... ... ... | 141 Harley Street, London, W. |
| Jones, W. R., M.D., C.M. Glasg. ... ... ... | Senny Bridge, Breconshire. |
| Jones, W. W., M.B., C.M. Ed. ... ... ... | 47 Wellington Street, Merthyr Tydvil. |
| | |
| *Kelly, T. B. ... ... ... ... ... ... ... ... | Eye Hospital, Bristol. |
| *Kemm, F. St. J.... ... ... ... ... ... ... ... | Worle. |
| Kempe, Arthur W., M.D. Brussels ... ... | 14 Southernhay, Exeter. |
| Kendall, Bernard C.... ... ... ... ... ... | Elm Grove, Penally, Pembrokeshire. |
| Kennedy, W. P., M.D., B.Ch., B.A. Dub. ... | High Street, Lydney. |
| *King, Preston, M.D. Cantab ... ... ... ... | 29 Gay Street, Bath. |
| Kinneir, R. ... ... ... ... ... ... ... ... | The Tower House, Malmesbury. |
| | |
| *Lace, F. ... ... ... ... ... ... ... ... ... | 5 Paragon, Bath. |
| *Lane, Hugh ... ... ... ... ... ... ... ... | 11 Circus, Bath. |
| *Lansdown, F. Poole ... ... ... ... ... ... | 19 White Ladies Road, Clifton. |
| *Lansdown, R. G. P., M.B., B.S. Dur. ... ... | 39 Oakfield Road, Clifton. |
| Lauwers, Dr. .. ... ... ... ... ... ... ... | Courtrai, Belgium. |
| *Lawrence, A. E. Aust, M.D. C.M. Aberd. . | 19 Richmond Hill, Clifton. |
| *Lawrence, Henry ... ... ... ... ... ... | 19 Park Row, Bristol. |
| Lawrie, J. Macpherson, M.D., C.M. Glasg. | Greenhill, Weymouth. |
| Leach, J. Comyns, M.D. Dur., B.Sc. Lond. . | Sturminster Newton. |
| *Lees, E. Leonard, M.D., C.M. Ed. ... ... | Redland Road, Bristol. |
| Leigh, W. W. ... ... ... ... ... ... ... ... | Treharris, Glamorganshire. |
| Lewellyn, John ... ... ... ... ... ... ... | Caerphilly. |
| *Lidderdale, James ... ... ... ... ... ... | Henley-on-Thames. |
| *Ligertwood, Walter ... ... ... ... ... ... | Royal Infirmary, Bristol. |
| Lilley, G. Herbert, M.D. Brussels ... ... | H.M. Convict Prison, Portland. |
| Lindsay, James A., M.D., M.Ch., M.A. Roy. Univ. I.... ... ... ... ... ... ... ... ... | 37 Victoria Place, Belfast. |
| Lister, Sir Joseph, Bart., F.R.S. ... ... ... | 12 Park Crescent, London, N.W. |
| Lloyd, Walter E. ... ... ... ... ... ... | 60 York Road, Bristol. |
| *Lowe, T. Pagan ... ... ... ... ... ... ... | 16 Circus, Bath. |
| Luckham, L. S. ... ... ... ... ... ... ... | 16 Harcourt Terrace, Salisbury. |
| Lucy, Reginald H., M.B., C.M. Ed. ... ... | 9 Princess Square, Plymouth. |
| | |
| Macdonald, J. A., M.D., M.Ch. Roy. Univ. I. | 19 East Street, Taunton. |
| Mac Donald, P. W., M.D., C.M. Aberd. ... | County Asylum, Dorchester. |
| Mac Ilwaine, S. W. ... ... ... ... ... ... | South Petherton, Somersetshire. |
| *Mackay, H. J., M.B., C.M. Ed. ... ... ... | 10 Long Street, Devizes. |
| Mackie, J., M.B., LL.D. Aberd., C.M.G. ... | Alexandria, Egypt. |
| Mackinnon, Charles, M.B., C.M., M.A. Glasg. | Dyer Street, Cirencester. |
| Maclean, Ewen J., M.D., C.M. Ed. ... ... | 51 Linden Gardens, London, W. |

### LIST OF SUBSCRIBERS.

| | |
|---|---|
| Maclean, J. Campbell, M.B., C.M Ed. | Swindon House, Swindon, Wiltshire. |
| *Macleod, H. W. G., M.B., C.M. Ed. | 23 Victoria Square, Clifton. |
| *McCrea, P. W., M.D., B.Ch., B.A., B.A.O., B.A. Dub. | 19 Portland Square, Bristol. |
| Macready, J. | 51 Queen Anne Street, London, W. |
| Marsh, O. E. Bulwer | Parkdale, Newport, Mon. |
| Marshall, Arthur L., M.B., B.A. Cantab. | 56 Rectory Road, London, N. |
| *Marshall, Henry, M.D. Ed., F.R.S.E. | 28 Caledonia Place, Clifton. |
| *Martin, E. F., M.B. Ed. | Victoria House, Weston-super-Mare. |
| Mason, S. B. | 12 Park Terrace, Pontypool. |
| Mason, William | Sedgemoor Cottage, St. Austell. |
| *Matthews, Charles E. | 51 Pembroke Road, Clifton. |
| Mayor, T. O. | 40 Apsley Road, Clifton. |
| McElfatrick, John | The Chantry, Mere, Wiltshire. |
| McNicoll, John | High Street, Poole. |
| Meredith, John, M.D. Ed. | Wellington, Somersetshire. |
| Metford, J. Seymour | 34 West Mall, Clifton. |
| *Millard, W. J. Kelson, M.D. St. And. | Stoke Bishop. |
| Milne, John, M.B., C.M. Aberd. | Hill Avenue, Bristol. |
| Milward, James, M.D. Brussels | 54 Charles Street, Cardiff. |
| *Mole, Harold F. | 5 Meridian Place, Clifton. |
| Monckton, William | Portishead. |
| Mouat-Biggs, C. E. F. | 2 Fauconberg Villas, Cheltenham. |
| Morgan, Frederick | Uffculme. |
| Morgan, John | Langport, Somersetshire. |
| *Morgan, T. Whitworth S. | Pill. |
| *Morton, C. A. | 24 St. Paul's Road, Clifton. |
| Munden, Charles | Ilminster. |
| Murray, William, M.B., C.M. Ed. | Staple Hill, Gloucestershire. |
| *Myles, G. T. | 3 Portland Square, Bristol. |

| | |
|---|---|
| Napier, T. W. A., M.D., C.M. Aberd. | Church Street, Egremont, Cheshire. |
| Neale, B. G. | 51 Upper Belgrave Road, Clifton. |
| Nell, Richard F. | 33 Windsor Terrace, Penarth. |
| *Nevill, W. N., M.D., B.Ch., B.A. Dub. | Southville, Bristol. |
| *Newman, Charles | 156 Cheltenham Road, Bristol. |
| *Newnham, W. H. C., M.B., M.A. Cantab. | St. Paul's Road, Clifton. |
| Nicholson, T. D., M.D., C.M. Ed. | 2 White Ladies Road, Clifton. |
| Norgate, R. H. | County Asylum, Maidstone. |
| Norman, J. H. | Goodleigh, Winkleigh, Devonshire. |
| *Norton, J. A., M.D., C.M. Aberd. | 65 Redcliff Hill, Bristol. |

| | |
|---|---|
| O'Brien, Cornelius | Down House, Whitehall, Bristol. |
| Odell, William | Ferndale, Torquay. |
| *Ogilvy, Alexander, M.D., B.Ch., B.A. Dub. | 20 Pembroke Road, Clifton. |
| Openshaw, E. Hyde | Barrules, Cheddar. |
| *Ormerod, Henry | Westbury-on-Trym. |
| *Ormerod, H. Lawrence, M.B., B.Ch., B.A.O., R.U.I. | Royal Infirmary, Bristol. |

| | |
|---|---|
| Page, G. Shepley | 78 Old Market Street, Bristol. |
| *Parker, George, M.D., M.A. Cantab. | 14 Pembroke Road, Clifton. |
| Parry, J. H. | 199 Cheltenham Road, Bristol. |
| Parsons, Francis J. C. | King Square, Bridgwater. |
| Paterson, Donald Rose, M.D., C.M. Ed. | 18 Windsor Place, Cardiff. |

### LIST OF SUBSCRIBERS.

| | |
|---|---|
| Peake, Arthur W. | 1 Bridewell Street, Bristol. |
| Peake, G. Arthur | Rodney Place, Cheltenham. |
| *Penny, F. | North Devon Infirmary, Barnstaple. |
| *Penny, W. J. | Richmond Park Road, Clifton. |
| Pentland, Young J. | 11 Teviot Place, Edinburgh. |
| Peskett, A. W. C., M.B., B.C., M.A. Cantab. | Burnham, Somersetshire. |
| Phelps, W. Harford G., M.D. Aberd. | Beachfield, Weston-super-Mare. |
| Phillips, Edward Picton | High Street, Haverfordwest. |
| Phillips, J. A. | 72 Carlisle Street, Cardiff. |
| Phillips, Sutherland Rees, M.D., M.Ch. Roy. Univ. I. | St. Ann's Heath, Virginia Water, Surrey. |
| *Pickering, C. F. | 12 Berkeley Square, Bristol. |
| *Pinch, A. E. H. | 53 Oakfield Road, Clifton. |
| Pippette, Walter | Dawes Road, London, S.W. |
| Plaister, W. H. | High Road, Tottenham. |
| Pollard, Reginald, M.B. Dur. | Southlands, Torquay. |
| *Pountney, W. E., M.D., C.M. Ed. | 33a White Ladies Road, Bristol. |
| Powell, J. J., M.D., Lond. | 2 Portland Square, Bristol. |
| Powell, William, M.B. Lond. | Hill Garden, Torquay. |
| Price, R. G., M.D. Dur. | Carmarthen. |
| Price, William, M.B. Lond. | 40 Park Place, Cardiff. |
| *Prichard, Arthur W. | 6 Rodney Place, Clifton. |
| *Prichard, Augustin, M.D. Berlin | 4 Chesterfield Place, Clifton. |
| *Prichard, J. E., M.B., B.A. Oxon. | 243 Cheltenham Road, Bristol. |
| Pring, Arthur, B.A. Cantab. | Clyde Road, Bristol. |
| *Prowse, Arthur B., M.D. Lond. | 5 Lansdown Place, Clifton. |

| | |
|---|---|
| Randolph, Charles | St. Michael's, Milverton, Somersetshire. |
| *Rattray J. M., M.D., C.M., M.A. Aberd. | Frome. |
| Redwood, T. Hall, M.D., C.M., M.A. Dur. | The Lawn, Rhymney, Monmouthshire. |
| Rees, Alfred | 46 Loudoun Square, Cardiff. |
| Rendall, John | Forestside, Lymington. |
| *Richardson, W., M.D. St. And. | 1 Belgrave Villas, Cotham Grove, Bristol. |
| Riddell, Andrew W. | St. Mary Bourne. |
| Robathan, G. B. | The Grove, Risca. |
| Roberts, Frederick T., M.D., B.Sc. Lond. | 102 Harley Street, London, W. |
| Roderick, Sydney J., M.B., C.M. Ed. | Llanelly. |
| Rodgers, John W., M.B., C.M. Ed. | Redfield, Gloucestershire. |
| *Rogers, B. M. H., M.D., B.Ch., B.A. Oxon. | 11 York Place, Clifton. |
| *Ross, R. A. | Lodway Villa, Pill. |
| *Rossiter, George F., M.B. Lond. | Cairo Lodge, Weston-super-Mare. |
| *Roué, W. Barrett, M.D., M.S. Dur. | 2 Buckingham Place, Clifton. |
| Routh, R. Henry F. | Bridgewater. |
| Row, F. Everard | 9 Tamar Terrace, Devonport. |
| *Roxburgh, Robert, M.B. Ed. | 1 Victoria Buildings, Weston-super-Mare. |
| *Rudge, C. King | 145 White Ladies Road, Bristol. |
| *Rudge, H. T. | 5 Colston Parade, Bristol. |
| Ruffer, M. Armand, M.D., B.S., M.A. Oxon. | 5 York Terrace, London, N.W. |
| Rutherford, H. T. | Park Street, Taunton. |

| | |
|---|---|
| Sandoe, J. W., M.D., B.S. Dur. | Broad Clyst. |
| Sawyer, J. A. F. | Albert Road, Clevedon. |
| Scimonelli, Sig^ra. Cruce | Naples. |
| Scott, James, M.B., C.M. Ed. | 24 Gaolgate Street, Stafford. |
| Scott, Richard J. H. | 28 Circus, Bath. |
| Searle, George C. | Brixham, Devonshire. |

### LIST OF SUBSCRIBERS.

| | |
|---|---|
| Searle, Richard Burford ... ... ... ... | Bexley, Dartmouth. |
| *Semple, H. F. ... ... ... ... ... ... ... ... ... | Budleigh Salterton, Devonshire. |
| Seward, W. J., M.B. Lond. ... ... ... ... | Asylum, Colney Hatch, London, N. |
| *Shaw, J. E., M.B., C.M. Ed. ... ... ... ... | 23 Caledonia Place, Clifton. |
| Shaw, Jesse, M.D. ... ... ... ... ... ... ... | Fort Beaufort, Cape Colony. |
| Sheen, A., M.D. St. And. ... ... ... ... ... | 23 Newport Road, Cardiff. |
| *Sheppard, E. J. ... ... ... ... ... ... ... ... | 11 Richmond Hill, Clifton. |
| *Shorland, Walter E. ... ... ... ... ... ... | Cecil Road, Bournemouth. |
| Shortridge, Thomas Wood, M.D. Brussels | Honiton. |
| Simons, C. E. G., M.B., C.M. Aberd. ... ... | The Hollies, Merthyr Tydvil. |
| *Sinclair, David, M.B.,C.M. Aberd. ... ... | 6 Whitehall Yard, London, S.W. |
| Sincock, J. Bain ... ... ... ... ... ... ... ... | Friarn Street, Bridgwater. |
| Skelton, Henry, M.D. St. And. ... ... ... | Downend, Gloucestershire. |
| *Skerritt, E. Markham, M.D., B.S., B.A. Lond. | Richmond Hill, Clifton. |
| Skinner, George H. ... ... ... ... ... ... ... | Kilmore, Victoria, Australia. |
| *Skinner, Stephen, M.B., C.M. Aberd. ... | Tranent Lawn, Clevedon. |
| *Smith, G. Munro ... ... ... ... ... ... ... ... | 28 Alma Road, Clifton. |
| *Smith, J. Greig, M.B., C.M., M.A. Ab., F.R.S.E. | 16 Victoria Square, Clifton. |
| Smith, Rev. Reginald, M.A. Cantab ... ... | 9 Richmond Hill, Clifton. |
| *Smith, R. Shingleton, M.D., B.Sc. Lond. | Clifton Park, Clifton. |
| Smith, W. Alexander, M.B., M.A. Oxon. ... | Newport, Essex. |
| Snell, Simeon ... ... ... ... ... ... ... ... | 17 Eyre Street, Sheffield. |
| Society, Cardiff Medical (*Hon. Sec.*, Dr. D. R. Paterson). | |
| Society, Plymouth Medical (*Hon. Lib.*, C. E. R. Rendle). | |
| Society, Torquay Medical (*Hon. Sec.*, G. Young Eales). | |
| Solly, Reginald V., M.B., B.S. Lond. ... | 7 Dix's Field, Exeter. |
| Solly, S. E. ... ... ... ... ... ... ... ... ... | Colorado Springs, Colorado, U.S.A. |
| Somer, James ... ... ... ... ... ... ... ... ... | Broad Clyst. |
| Soutar, James Greig, M.B., C.M. Ed. ... ... | Barnwood House, Gloucester. |
| *Spencer, W. H., M.D., M.A. Cantab. ... ... | 11 Warrior Square, St. Leonard's-on-Sea. |
| Stamp, W. D. ... ... ... ... ... ... ... ... ... | Ridgeway House, Plympton. |
| Statham, R. Whiteside ... ... ... ... ... | Cheddar, Somersetshire. |
| *Steele, Charles, M.D. Dur. ... ... ... ... | 17 Richmond Hill, Clifton. |
| Steele-Perkins, E. B. ... ... ... ... ... ... | 1 Hill's Buildings, Exeter. |
| Stevens, W. H. ... ... ... ... ... ... ... ... | Zetland Road, Bristol. |
| Stoddart, F. Wallis ... ... ... ... ... ... ... | 1 Park Street, Bristol. |
| *Swain, James, M.D., M.S. Lond. ... ... ... | 7 Buckingham Place, Clifton. |
| *Swayne, J. G., M.D. Lond. ... ... ... ... | 74 Pembroke Road, Clifton. |
| *Swayne, S. H. ... ... ... ... ... ... ... ... ... | 129 Pembroke Road, Clifton. |
| *Swayne, W. Carless, M.D. Lond. ... ... ... | St. Paul's Road, Clifton. |
| Swete, H. Lawton ... ... ... ... ... ... ... | Fishguard, Pembrokeshire. |
| Symonds, H. P. ... ... ... ... ... ... ... ... | 35 Beaumont Street, Oxford. |
| Symons, John ... ... ... ... ... ... ... ... ... | Buriton House, Penzance. |

| | |
|---|---|
| Taylor, C. Bell, M.D. Edin. ... ... ... ... | 9 Park Row, Nottingham. |
| *Taylor, James ... ... ... ... ... ... ... ... | 36 Alma Road, Clifton. |
| Taylor, Thomas Henry ... ... ... ... ... | Thornbury, Gloucestershire. |
| Thomas, A. Garrod, M.D., C.M. Ed. ... ... | Clytha Park, Newport, Mon. |
| Thomas, J. Lynn ... ... ... ... ... ... ... ... | 28 Charles Street, Cardiff. |
| Thomas, John T. ... ... ... ... ... ... ... ... | Brynhyfryd, Chelston, Torquay. |
| Thomas, Rowland ... ... ... ... ... ... | The Cwfr, St. Clears, Carmarthenshire. |
| Thomas, W. H. ... ... ... ... ... ... ... ... ... | Maesteg, Glamorganshire. |
| Thompson, W. C. ... ... ... ... ... ... ... ... | 189 Cheltenham Road, Bristol. |
| Thomson, Eustace B., M.D., C.M. Glasg. ... | Albany Place, Plymouth. |
| Thorne, G. Leworthy, M.D. Aberd. ... ... | Cheriton Fitzpaine. |
| *Tivy, W. J. ... ... ... ... ... ... ... ... ... | 8 Lansdown Place, Clifton. |
| Tolputt, Percy T. ... ... ... ... ... ... ... | Weaver Lane, Northwich. |

### LIST OF SUBSCRIBERS.

| | |
|---|---|
| Tosswill, Louis H., M.B., B.A. Cantab. ... | 28 West Southernhay, Exeter. |
| Tracey, H. E., M.B. Lond. ... ... ... ... ... | Willand. |
| Tribe, Ethel N., M.B. Lond. ... ... ... ... | 155 White Ladies Road, Bristol. |
| Trotter, Leslie B., M.D., C.M. Ed. ... ... | Coleford, Gloucestershire. |
| Tyley, R. P., M.D. St. And. ... ... ... ... ... | Wedmore. |

| | |
|---|---|
| Vachell, Herbert R., M.D., C.M. Aberd.... | 18 Newport Road, Cardiff. |
| Vereker, R. H.... ... ... ... ... ... ... ... | Eastleigh, Curry Rivel. |

| | |
|---|---|
| Wade, A. Law, M.D., B.A. Dub. ... ... ... | County Asylum, Wells, Somersetshire. |
| Wade, Charles H., M.A. Oxon. ... ... ... | Stanfield, Torquay. |
| *Waldo, Henry, M.D., C.M. Aberd. ... ... | 19 Pembroke Road, Clifton. |
| *Walker, Cyril H., M.B., B.A. Cantab.... ... | St. Paul's Road, Clifton. |
| Wallace, Rev. Canon, M.A. Oxon. ... ... | 3 Harley Place, Clifton. |
| *Wallace, John ... ... ... ... ... ... ... | 1 Oriel Terrace, Weston-super-Mare. |
| Walsh, Leslie H. ... ... ... ... ... ... ... | Royal United Hospital, Bath. |
| Walter, William Henry, M.D. Brussels ... | Winslow. |
| Ward, J. L. W. ... ... ... ... ... ... ... | Victoria Street, Merthyr Tydvil. |
| *Wathen, J. Hancocke ... ... ... ... ... | 16 York Place, Clifton. |
| Watson, J. Adam ... ... ... ... ... ... ... | 39 Dennington Park, London, N.W. |
| Watters, George T. B., M.D., C.M. Ed. ... | Stonehouse, Gloucestershire. |
| Waugh, Alexander ... ... ... ... ... ... | Midsomer Norton. |
| Weatherly, Lionel A., M.D., C.M. Aberd. | Bailbrook House, Bath. |
| Webber, William W.... ... ... ... ... ... | Crewkerne. |
| *Webster, Thomas ... ... ... ... ... ... | Redland Hill, Bristol. |
| *Wedmore, C. Ernest, M.B., M.A. Cantab. | Stoke Bishop. |
| Wethered, Frank J., M.D. Lond. ... ... | 34 Queen Anne Street, London, W. |
| *Whicher, A. H. ... ... ... ... ... ... ... | Midsomer Norton. |
| Whyte, Andrew, M.D., C.M. Aberd. ... ... | Honddû House, Brecon. |
| Wickham, W. ... ... ... ... ... ... ... ... | Hill House, Tetbury. |
| Wigan, C. A., M.D. Dur. ... ... ... ... ... | Portishead. |
| Wigmore, J., M.D. Roy. Univ. I. ... ... ... | Twerton-on-Avon. |
| Wilcox, Henry, M.B. Aberd. ... ... ... ... | Fleet, Hampshire. |
| Wilde, Rowland ... ... ... ... ... ... ... | Park House, Weston-super-Mare. |
| *Wilding, James, M.D. Dur. ... ... ... ... | Sydenham Road, Bristol. |
| Willcox, R. Lewis ... ... ... ... ... ... | Warminster. |
| Willett, George G. D. ... ... ... ... ... | Keynsham, Somersetshire. |
| Williams, Charles ... ... ... ... ... ... | 116 Derby Road, Bootle, Liverpool. |
| Williams, D. J. ... ... ... ... ... ... ... | Greenfield House, Llanelly. |
| Williams, H. Egerton ... ... ... ... ... | The Mount, Newport, Mon. |
| Williams, John, M.D.... ... ... ... ... ... | 20 Windsor Place, Cardiff. |
| *Williams, Lionel H. ... ... ... ... ... ... | Thornbury, Gloucestershire. |
| *Williams, P. Watson, M.D. Lond. ... ... | 2 Lansdown Place, Clifton. |
| Williams, Peter ... ... ... ... ... ... ... | Ferryside, Carmarthenshire. |
| *Windsor-Aubrey, H. W. ... ... ... ... ... | Coronation Road, Bristol. |
| Winterbotham, L. ... ... ... ... ... ... | Bays Hill, Cheltenham. |
| *Wintle, Colston ... ... ... ... ... ... ... | Belgrave Road, Bristol. |
| Woodford, E. Russell, M.D., C.M. Ed. ... | 4 Prospect Terrace, Ventnor. |
| Woodhead, G. Sims, M.D., C.M. Ed. ... ... | 1 Nightingale Lane, London, S.W. |
| Woods, Frank ... ... ... ... ... ... ... | Timsbury. |
| Woollcombe, Walter L. ... ... ... ... ... | 16 Lockyer Street, Plymouth. |

| | |
|---|---|
| Yelf, Leonard K., M.D. St. And. ... ... ... | Moreton-in-Marsh. |

### Exchange-List.

### AMERICA.

### ARGENTINE REPUBLIC.

*Weekly.*

*Semana Médica.* Buenos Ayres: J. A. Manzano.

### CANADA.

*Monthly.*

*The Canadian Practitioner.* Toronto: The J. E. Bryant Company.
*The Montreal Medical Journal.* Montreal: Gazette Printing Company.

### MEXICO.

*Twice a month.*

*Gaceta Médica.* Mexico: Avenida 2 Oriente, 726.

### PERU.

*Monthly.*

*La Crónica Médica.* Lima: 24 Calle de Bodegones.

### UNITED STATES.

*Weekly.*

*Boston Medical and Surgical Journal.* Boston: Damrell & Upham.
*Medical Record.* New York: William Wood & Co.
*Medical Review.* St. Louis: 914 Locust Street.
*The Cincinnati Lancet-Clinic.* Cincinnati: 199 West 7th Street.
*The Journal of the American Medical Association.* Chicago: 86 Fifth Avenue.
*The Medical and Surgical Reporter.* Philadelphia 147-149-151 North Tenth Street.
*The Medical News.* Philadelphia: Lea Bros. & Co.
*The New York Medical Journal.* New York: D. Appleton & Co.

*Fortnightly.*

*The American Practitioner and News.* Louisville: John P. Morton and Company.

*Twice a month.*

*The Medical Age.* Detroit: George S. Davis.

*Monthly.*

*Archives of Pediatrics.* New York: Bailey & Fairchild.
*Buffalo Medical and Surgical Journal.* Buffalo: Times Printing House.
*Bulletin of the Johns Hopkins Hospital.* Baltimore: The Johns Hopkins Press.
*California Medical Journal.* California: 1422 Folsom Street.
*Index Medicus.* Detroit: George S. Davis.
*International Medical Magazine.* Philadelphia: International Medical Magazine Company.
*Journal of Cutaneous and Genito-Urinary Diseases.* New York: D. Appleton & Co.
*Occidental Medical Times.* Sacramento: D. Johnston & Co.
*The American Journal of Obstetrics and Diseases of Women and Children.* New York: Wm. Wood & Co.
*The American Journal of Ophthalmology.* St. Louis: J. H. Chambers & Co.
*The American Journal of the Medical Sciences.* Philadelphia: Lea Brothers & Co.
*The Journal of Nervous and Mental Disease.* New York: 25 West 45th Street.
*The Post-Graduate.* New York: 2nd Avenue and 20th Street.
*The Saint Louis Medical and Surgical Journal.* Saint Louis: St. Louis Medical and Surgical Journal Publishing Company.
*The Therapeutic Gazette.* Detroit: George S. Davis.
*The Universal Medical Journal.* Philadelphia: The F. A. Davis Company.
*University Medical Magazine.* Philadelphia: University of Pennsylvania Press.

*Quarterly.*

*Mathews' Medical Quarterly.* Louisville: John [illegible] Morton & Company.

*The Alienist and Neurologist.* St. Louis: Ev. [illegible] Carreras.

*Yearly.*

*Annual of the Universal Medical Sciences.* Phila- delphia: The F. A. Davis Company.
*Transactions of the American Surgical Associati[illegible]* Philadelphia: William J. Dornan.
*Transactions of the New York Academy of Medici[illegible]* New York: Stettiner, Lambert & Co.

### ASIA.

### CHINA.

*Quarterly.*

*The China Medical Missionary Journal.* Shanghai: The American Presbyterian Mission Press.

### INDIA.

*Monthly.*

*The Indian Medico-Chirurgical Review.* Bombay: [illegible] Rao & Co.

### JAPAN.

*Monthly.*

*The Sei-I-Kwai Medical Journal.* Tokio: 6 Shi[illegible] Sakana Chō, Kyōbashi Ku.

### AUSTRALASIA.

### AUSTRALIA.

*Monthly.*

*The Australasian Medical Gazette.* Sydney: L. Bruck.

*Quarterly.*

*Intercolonial Quarterly Journal of Medicine and Surgery.* Melbourne: Stillwell & Co.

### NEW ZEALAND.

*Quarterly.*

*The New Zealand Medical Journal.* Dunedin: J. Wilkie & Co.

### EUROPE.

### AUSTRIA.

*Weekly.*

*Wiener klinische Wochenschrift.* Vienna: Wilhelm Braumüller.

### BELGIUM.

*Monthly.*

*Bulletin de l'Académie royale de médecine de Belgique.* Brussels: F. Hayez.

### BRITISH ISLES.

*Weekly.*

*British Medical Journal.* London: 429 Strand.
*Medical Press and Circular.* London: A. A. Tindall.
*The Clinical Journal.* London: E. Knight.
*The Hospital.* London: 428 Strand.
*The Lancet.* London: 423 Strand.
*The Sanitary Record.* London: Newspaper Distri- buting Agency.

*Monthly.*

*Clinical Sketches.* London: Smith, Elder & Co.

*The Birmingham Medical Review.* Birmingham: Hall & English.

*The British Journal of Dermatology.* London: H. K. Lewis.

*The Journal of Laryngology, Rhinology, and Otology.* London: F. J. Rebman.

*The Medical Chronicle.* Manchester: John Heywood.

*The Medical Magazine.* London: Southwood, Smith and Co.

*The Practitioner.* London: Cassell & Co. Limited.

*Edinburgh Medical Journal.* Edinburgh: Oliver and Boyd.

*The Dublin Journal of Medical Science.* Dublin: Fannin & Co.

*Quarterly.*

*Archives of Surgery.* London: J. & A. Churchill.

*The Asclepiad.* London: Longmans, Green & Co.

*The British Gynæcological Journal.* London: John Bale & Sons.

*The Quarterly Medical Journal.* Sheffield: Pawson and Brailsford.

*Half-yearly.*

*The Liverpool Medico-Chirurgical Journal.* Liverpool: Medical Institution.
*The Retrospect of Medicine.* London: Simpkin & Co. Limited.

*Yearly.*

*Clinical Society's Transactions.* London: Longmans, Green & Co.
*Guy's Hospital Reports.* London: J. & A. Churchill.
*King's College Hospital Reports.* London: Adlard & Son.
*Medico-Chirurgical Transactions.* London: Longmans, Green & Co.
*Obstetrical Society Transactions.* London: 20 Hanover Square.
*Ophthalmological Transactions.* London: J. & A. Churchill.
*St. Bartholomew's Hospital Reports.* London: Smith, Elder & Co.
*St. Thomas's Hospital Reports.* London: J. & A. Churchill.
*The Medical Annual.* Bristol: John Wright & Co.
*The Medical Society's Transactions.* London: Harrison & Sons.
*The Westminster Hospital Reports.* London: J. & A. Churchill.
*The Year-Book of Treatment.* London: Cassell & Company, Limited.
*Transactions of the Royal Academy of Medicine in Ireland.* Dublin: Fannin & Co.
*Year-Book of Scientific and Learned Societies.* London: C. Griffin & Co.

*Occasionally.*

*The Directory of Second-hand Booksellers.* Rochdale: James Clegg.
*Transactions of the West London Medico-Chirurgical Society.* London: Baillière, Tindall & Cox.

DENMARK.

*Weekly.*

*Hospitalstidende.* Copenhagen: Jacob Lund.

FRANCE.

*Three times a week.*

*Gazette des hôpitaux.* Paris: 4 Rue de l'Odéon.

*Weekly.*

*Bulletin de l'Académie de médecine.* Paris: G. Masson.

*Gazette hebdomadaire des Sciences Médicales de Bordeaux.* Bordeaux: P. Cassignol.

*Gazette des hôpitaux de Toulouse.* Toulouse: 3 Avenue Lafayette.

*La France médicale.* Paris: 14 Rue des Pyramides.

*Le Progrès médical.* Paris: 14 Rue des Carmes.

*L'Union médicale.* Paris: Octave Doin.

*Twice a month.*

*Gazette de Gynécologie.* Paris: 12 Rue de Châteaudun.

*La Revue médicale.* Paris: F. Sallé.

*Revue de Thérapeutique médico-chirurgicale.* Paris: 6 Rue Gît-le-Cœur.

*Revue internationale de Médecine et de Chirurgie pratiques.* Paris: 37 Rue Taitbout.

*Fortnightly.*

*Le Nord médical.* Lille: 54 Rue Basse.

*Monthly.*

*Archives cliniques de Bordeaux.* Bordeaux: Feret et Fils.

*Archives de Neurologie.* Paris: 14 Rue des Carmes.

*Revue de Laryngologie, d'Otologie et de Rhinologie.* Paris: Octave Doin.

*Revue générale d'Ophtalmologie.* Paris: G. Masson.

*Revue des Instruments de chirurgie.* Paris: 34 Rue de Seine.

*Revue médico-chirurgicale des Maladies des Femmes.* Paris: 45 Boulevard Malesherbes.

*Revue obstétricale et gynécologique.* Paris: 35 Boulevard Haussmann.

*Quarterly.*

*Bulletins et Mémoires de la Société de Médecine et de Chirurgie pratiques.* Paris: 28 Rue Serpente.

*Half-yearly.*

*Travaux d'Electrothérapie gynécologique.* Paris: Société d'Editions Scientifiques.

GERMANY.

*Weekly.*

*Centralblatt für Chirurgie.* Leipzig: Breitkopf & Härtel.

*Centralblatt für Gynäkologie.* Leipzig: Breitkopf & Härtel.

*Centralblatt für innere Medicin.* Leipzig: Breitkopf & Härtel.

*Münchener Medicinische Wochenschrift.* Munich: J. F. Lehmann.

*Monthly.*

*Zeitschrift für Krankenpflege.* Berlin: H. Kornfeld.

GREECE.

*Weekly.*

**Γαληνὸς.** Athens: N. G. Passare.

HOLLAND.

*Weekly.*

*Nederlandsch Tijdschrift voor Geneeskunde.* Amsterdam: F. van Rossen.

ITALY.

*Weekly.*

*Gazzetta Medica Lombarda.* Milan: 33 Via Spiga.

*Twice a month.*

*Giornale Internazionale delle Scienze Mediche.* Naples. Enrico Detken.

*Monthly.*

*Annali di Ostetricia e Ginecologia.* Milan: Lodovico Felice Cogliati.

NORWAY.

*Monthly.*

*Norsk Magazin for Lægevidenskaben.* Christiania: Det Steenske Bogtrykkeri.

PORTUGAL.

*Weekly.*

*A Medicina Contemporanea.* Lisbon: José Antonio Rodrigues.

ROUMANIA.

*Twice a month.*

*Spitalul.* Bucharest: Thoma Basilescu.

*Monthly.*

*La Roumaine Médicale.* Bucharest: L'Institut de Bactériologie.

RUSSIA.

*Weekly.*

*Medycyna.* Warsaw: 80 Allée de Jerusalem.
*Vrach.* St. Petersburg: C. Ricker.
*St. Petersburger Medicinische Wochenschrift.* St. Petersburg: Carl Ricker.

*Monthly.*

*Finska Läkaresällskapets Handlingar.* Helsingfors: Helsingfors Central-Tryckeri.

SPAIN.

*Weekly.*

*El Siglo Médico.* Madrid: Magdalena, 36.

*Twice a month.*

*Revista de Ciencias Médicas.* Barcelona: 9 Calle Fortuny.

SWEDEN.

*Quarterly.*

*Nordiskt Medicinskt Arkiv.* Stockholm: P. A. Norstedt & Söner.

TURKEY.

*Twice a month.*

*Gazette Médicale d'Orient.* Constantinople: A. Christidis.

### *PUBLICATIONS RECEIVED.*

From ADLARD & SON, London:
*Diphtheria Chart.*

From E. W. ALLEN, London:
*Food and Sanitation.* Vol. VI., No. 130. Feb. 2nd, 1895.

From ALLEN & HANBURYS, London:
*Izal.* [N.D.]

From THE AMERICAN THERAPIST PUBLISHING COMPANY, New York:
*The American Therapist.* Vol. III., Nos. 6, 7. Dec., 1894, Jan., 1895.

From BAILLIÈRE, TINDALL & COX, London:
*Bread, Bakehouses, and Bacteria.* By F. J. WALDO, M.A., M.D. and DAVID WALSH, M.B., C.M. 1895.
*Diphtheria and its Treatment.* By BROWNLOW R. MARTIN, A.B., M.B. Second Edition, 1895.

From CASSELL & COMPANY, LIMITED, London:
*Diseases of the Ear.* By A. MARMADUKE SHEILD, M.B. 1895.

From THE CHURCH DEFENCE INSTITUTION, London:
*The National Church.* Vol. XXIV., Nos. 278—280. Jan.—Mar., 1895.

From J. & A. CHURCHILL, London:
*Syphilis.* By ALFRED COOPER. Second Edition. Edited by EDWARD COTTERELL. 1895.
*Operating for Cataract.* By Surgeon-Captain G. H. FINK. 1894.
*Abdominal Tumours and Abdominal Dropsy.* By JAMES OLIVER, M.D. 1895.

From THE CHURCH MISSIONARY HOUSE, London:
*Medical Mission Quarterly.* No. 9. Jan., 1895.

From WILLIAM F. CLAY, Edinburgh:
*Tropical Diseases in Africa.* By R. W. FELKIN, M.D. 1895.

From COMPANIA SUD-AMERICANA DE BILLETES DE BANCO, Buenos Aires:
*Revista de la Sociedad Mědica Argentina.* Vol. III., No. 18. 1894.

From FERRIS & CO., Bristol:
*Pocket Therapeutic Notes.* Second Edition. 1894.

From "ILLUSTRATED LONDON NEWS" OFFICE, London:
*The English Illustrated Magazine.* Feb., Mar., 1895.
*The Album.* No. 1. Feb. 4th, 1895.

From JUDD & DETWEILER, Washington:
*Anatomy and Art.* By ROBERT FLETCHER, M.D. 1895.

From 723 KANSAS AVENUE, Topeka, Kansas:
*The Kansas Medical Journal.* Vol. VI., Nos. 49, 52. Dec. 8th, 29th, 1894. Vol. VII., Nos. 1, 2. Jan. 5th, 12th, 1895

From KNOLL & CIE, Ludwigshafen a Rh.:
*Die therapeutische Anwendung des Ferropyrins.* Von Dr. W. CUBASCH 1895. [Reprint.]

From H. K. LEWIS, London:

*An Introductory Lecture on the Public, the Profession, and Medical Charities.* By ROBERT BOXALL, M.D. 1894.

*The Prevention of Small-Pox.* By EDGAR M. CROOKSHANK, M.B. 1894.

*Pulse-Gauging* By GEORGE OLIVER, M.D. 1895.

*Atlas of the Nasal Cavity and Sinuses.* By Dr. A. ONODI. Translated from the Second Edition by ST. CLAIR THOMSON, M.D. 1895.

*The Aseptic Treatment of Wounds.* By Dr. C. SCHIMMELBUSCH. Translated from the Second German Edition by ALFRED THEODORE RAKE, M.B. 1894.

*Medical Nursing.* By the late JAMES ANDERSON, M.D. Edited by ETHEL F. LAMPORT. Second Edition. 1895.

*Diseases of the Eye.* By HENRY R. SWANZY, M.B., Fifth Edition Edited by LOUIS WERNER, M.B., B.Ch. 1895.

*Droitwich Brine Baths.* By W. H. TOMLINS. 1895.

*The Middlesex Hospital, W. Reports of the Medical and Surgical Registrars, and Pathologist, for the Year* 1893. 1894,

From PETER MÖLLER, London:

*Cod-Liver Oil and Chemistry.* By F. PECKEL MÖLLER, Ph.D. 1895.

From THE PACIFIC DRUGGIST PUBLISHING Co., San Francisco:

*Pacific Druggist and Physician.* Vol. V., No. 9. Jan. 31st, 1895.

From 63 QUEEN VICTORIA STREET, London:

*The Physiology and Therapy of Ichthyol.* 1895.

From JOHN MORGAN RICHARDS, London:

*Medical Reprints.* Dec., 1894,—Feb. 1895.

From ROSE & HARRIS, Bristol:

*Food for the Sick and Convalescent.* By MARY FEACHEM. 1895.

From W. B. SAUNDERS, Philadelphia:

*Notes on the Newer Remedies.* By DAVID CERNA, M.D., Ph.D. Second Edition. 1895.

*Laboratory Guide for the Bacteriologist.* By LANGDON FROTHINGHAM. 1895.

*Syllabus of Gynecology.* By J. W. LONG, M.D. 1895.

From THE SCIENTIFIC PRESS, LIMITED, London:

*Helps in Sickness and to Health.* By HENRY C. BURDETT. 1894.

From AUG. SIEGLE, London:

*The Therapist.* Vol. IV., No. 12. 15th Dec., 1894.

From SIMPKIN, MARSHALL, HAMILTON, KENT & Co. LTD., London:

*Wintering in Egypt.* By ARTHUR J. M. BENTLEY, M.D. and Rev. C. G. GRIFFINHOOFE, M.A. 1894.

From SMITH, ELDER & Co., London:

*The Sick Poor in Workhouses.* By A SPECIAL COMMISSION OF THE "BRITISH MEDICAL JOURNAL." First Series. 1895.

From EBENEZER SOUTHCOTT, London:

*The St. James's Budget.* May 18th, 1894.

From GEORGE TIEMANN & Co., New York:

*Tiemann's Reprints.* No. 12.

From WILLIAMS & NORGATE, London:

*Teratologia.* Vol. I., Nos. 3, 4. Oct., Dec., 1894. Vol. II., No. 1. Jan , 1895.

From JOHN WRIGHT & Co., Bristol:

*Urinary Surgery.* By E. HURRY FENWICK. 1894.

### MANUALS
FOR
### Students and Practitioners of Medicine

*Published by CASSELL & COMPANY.*

***A New and Original Work on Surgery.***

**In Two Vols., price 48s.**

### A System of Surgery

BY VARIOUS AUTHORS.

EDITED BY FREDERICK TREVES, F.R.C.S.,

*Surgeon to, and Lecturer on Surgery at, the London Hospital; Examiner in Surgery at the University of Cambridge.*

CONTENTS AND WRITERS IN VOL. I.

**Surgical Bacteriology.** By GERMAN SIMS WOODHEAD, M.D., *Director of the Laboratories of the Royal Colleges of Physicians (Lond.) and Surgeons (Eng.).*

**Inflammation.** By W. WATSON CHEYNE, F.R.S., F.R.C.S., *Surgeon to King's College Hospital; Professor of Surgery at King's College.*

**Suppuration.** By W. WATSON CHEYNE, F.R.S.

**Ulceration.** By W. WATSON CHEYNE, F.R.S.

**Gangrene.** By W. WATSON CHEYNE, F.R.S.

**Syncope and Shock.** By W. WATSON CHEYNE, F.R.S.

**Erysipelas.** By C. B. LOCKWOOD, F.R.C.S., *Assistant Surgeon to St. Bartholomew's Hospital; Surgeon to the Great Northern Hospital.*

**Pyæmia.** By C. B. LOCKWOOD, F.R.C.S.

**Tetanus and Tetany.** By C. B. LOCKWOOD, F.R.C.S.

**Wounds and Contusions.** By W. WATSON CHEYNE, F.R.S.

**Military Surgery.** By SURGEON-MAJOR DUNCAN, M.D., B.S.Lond., F.R.C.S.

**Burns and Scalds.** By C. B. LOCKWOOD, F.R.C.S.

**The Influence of Constitutional Conditions upon Injuries.** By THE EDITOR.

**Anæsthetics.** By FREDERIC W. HEWITT, M.D. Cantab., *Anæsthetist to, and Instructor in Anæsthetics at, the London Hospital.*

**Surgical Diseases due to Microbic Infection and Parasites.** By GEORGE HENRY MAKINS, F.R.C.S., *Assistant-Surgeon to St. Thomas's Hospital; Surgeon to the Evelina Hospital for Children.*

**Tuberculosis.** By THE EDITOR.

**Rickets.** By GEORGE HENRY MAKINS, F.R.C.S.

**Hæmophilia.** By THE EDITOR.

**Hysteria in its Surgical Relations.** By THE EDITOR.

**Syphilis and Gonorrhœa.** By JONATHAN HUTCHINSON, Junr., F.R.C.S., *Assistant-Surgeon to the London Hospital; Surgeon to the Lock Hospital.*

**Tumours.** By J. BLAND SUTTON, F.R.C.S., *Assistant-Surgeon to, and Lecturer on Anatomy at, the Middlesex Hospital.*

**Injuries and Diseases of Blood-Vessels.** By A. PEARCE GOULD, F.R.C.S., M.S. Lond., *Senior Assistant-Surgeon to the Middlesex Hospital.*

**Aneurysm.** By A. PEARCE GOULD, F.R.C.S.

**Injuries and Diseases of Lymphatics.** By J. HAMMOND MORGAN, F.R.C.S., *Lecturer on Clinical Surgery at Charing Cross Hospital.*

**Injuries and Diseases of Nerves.** By ANTHONY ALFRED BOWLBY, F.R.C.S., *Assistant-Surgeon to St. Bartholomew's Hospital.*

**Diseases of the Skin.** By JONATHAN HUTCHINSON, Junr., F.R.C.S.

**Fractures.** By STANLEY BOYD, F.R.C.S., *Surgeon to Charing Cross Hospital.*

**Diseases of Bones.** By H. H. CLUTTON, F.R.C.S., M.B. Cantab., *Surgeon to, and Lecturer on Surgery at, St. Thomas's Hospital.*

**Diseases of the Jaws.** By J. BLAND SUTTON, F.R.C.S.

***With Two Coloured Plates and Several Hundred Original Woodcut Illustrations by CHARLES BERJEAU, F.L.S., and others.***

CASSELL & COMPANY, LIMITED, *Ludgate Hill, London.*

In Two Vols., *price* £2 2s.

### A Manual of Operative Surgery.

By FREDERICK TREVES, F.R.C.S.,

*Surgeon to, and Lecturer on Anatomy at, the London Hospital; Member of the Board of Examiners at the Royal College of Surgeons.*

With 422 Illustrations.

VOL. I.

GENERAL PRINCIPLES — OPERATIONS UPON ARTERIES AND NERVES—AMPUTATIONS—EXCISIONS.

VOL. II.

PLASTIC SURGERY — OPERATIONS UPON THE NECK AND ABDOMEN — OPERATIONS ON HERNIA — OPERATIONS UPON THE BLADDER, SCROTUM, PENIS, AND RECTUM — OPERATIONS UPON THE HEAD AND SPINE, THORAX AND BREAST.

"The relative merits of different operations are sufficiently and aptly discussed. . . . The anatomy of organs and regions is given with all necessary detail, and, of course, with accuracy. . . . The majority of the illustrations are new and original, and they are admirable in design and execution—real helps to the reader. The feature of all others in this work which specially commends it, and in which it is in striking contrast to some manuals and text-books, is the pains Mr. Treves has taken to mention the work of other surgeons under their name. . . . This is a special and very praiseworthy feature of the book, and is the strongest evidence of the care taken in its preparation."—*The Lancet.*

---

### Difficult Labour.

A Guide to its Management. For Students and Practitioners. By G. Ernest Herman, M.B. Lond., F.R.C.P., Senior Obstetric Physician to the London Hospital; Physician to the General Lying-in Hospital, &c. &c. With 162 Illustrations. 12*s.* 6*d.*

*CONTENTS.*

What is Natural Labour?
Difficult Occipito-posterior Positions.
Face and Brow Presentations.
The Moulding of the Head.
Pelvic Presentations.
Transverse Presentations.
Prolapse of Extremities.
Anomalies of the Umbilical Cord.
Twins.
Malformed Children.
Abnormal Uterine Action.
The Common Forms of Contracted Pelvis.
The Results of Contracted Pelvis.
The Diagnosis of Pelvic Contraction.
The Mechanism of Labour with Contracted Pelvis.
Treatment of Labour with Contracted Pelvis.
The Rare Forms of Contracted Pelvis.
Slow Dilatation of the Soft Parts.
Labour complicated with Tumours.
Rupture of the Uterus.
The Injuries to the Genital Canal in Childbirth.
Hæmorrhage before Delivery.
Hæmorrhage after Delivery.
The Forceps.
Turning.
Operations for Lessening the Child's Size.
Cæsarian Section.
Symphysiotomy.
The Induction of Premature Labour.

CASSELL & COMPANY, Limited, *Ludgate Hill, London.*

Price 12s. 6d.

### Surgical Diseases of the Ovaries and Fallopian Tubes,

### *Including* TUBAL PREGNANCY.

**By J. BLAND SUTTON, F.R.C.S.,**

*Assistant-Surgeon to the Middlesex Hospital; Late Hunterian Professor, and Erasmus Wilson Lecturer, Royal College of Surgeons, England.*

With 118 Engravings and Five Coloured Plates.

"We have nothing but praise for the contents of the book."—*Edinburgh Medical Journal.*

"The whole Work is well and clearly written, and may be commended to all those who desire to have a sound and judicial guide to the present state of our knowledge in these branches of medicine."—*Practitioner.*

---

### Tumours, Innocent and Malignant:

Their Clinical Characters and Appropriate Treatment. By J. BLAND SUTTON, F.R.C.S. With 250 Engravings, and 9 Plates. 21*s.*

"An interesting and suggestive guide to the knowledge of a very important branch of pathology."—*Guy's Hospital Gazette.*

"The work is one which will enhance the reputation of its author as a surgeon and pathologist. It would give a poor conception of the book if we did not refer to the wealth and beauty of its illustrations . . . a work which must have entailed on its author the expenditure of infinite labour and patience, and which there can be little question will rank high among works of its class."—*The Lancet.*

"The paper is unusually thick, the type large and clear, and the three-fold index admirably conceived. In conclusion, we would say that the book is original in design, excellent in detail, and as interesting for present perusal as it will be for future reference."—*Medical Press.*

CASSELL & COMPANY, LIMITED, *Ludgate Hill, London.*

### The Student's Handbook of Surgical Operations.

By FREDERICK TREVES, F.R.C.S. With 94 Illustrations. (Abridged from the Author's "Manual of Operative Surgery.") *4th Thousand.* 7s. 6d.

---

Price 5s.

### FIRST LINES IN MIDWIFERY:

### *A GUIDE TO ATTENDANCE ON NATURAL LABOUR FOR MEDICAL STUDENTS AND MIDWIVES.*

**By G. ERNEST HERMAN, M.B. Lond., F.R.C.P.,**

*Obstetric Physician to the London Hospital, and Lecturer on Midwifery; Physician to the General Lying-in Hospital; Formerly Physician to the Eastern District of the Royal Maternity Charity; Examiner in Midwifery to the Royal College of Surgeons; Consulting Physician-Accoucheur to the Tower Hamlets Dispensary; Treasurer of, and late Examiner to, the Obstetrical Society of London.*

With 80 Illustrations.

"The drawings are well chosen from different Works on Obstetrics by recognised authorities. The paragraphs on the preparation of antiseptics for the lying-in chamber, and the passages on the feeding of infants, are alike of high excellence. The young practitioner will profit by them, as well as the student and the midwife. This manual is of considerable merit, and is likely to prove highly popular in London Schools and Lying-in Hospitals."—*British Medical Journal.*

"Dr. Herman has succeeded in producing a book admirably well fitted for the purposes he has had in view, the education of the midwife and the supplying of the undergraduate with a handy book on Obstetrics."—*Edinburgh Medical Journal.*

CASSELL & COMPANY, LIMITED, *Ludgate Hill, London.*

### MANUALS FOR STUDENTS OF MEDICINE

*Published by CASSELL & COMPANY.*

*NEW AND REVISED EDITION.*

### Hygiene and Public Health.

By B. ARTHUR WHITELEGGE, M.D., B.Sc. Lond., D.P.H. Camb.,

*Medical Officer of Health to the West Riding County Council, late Medical Officer of Health for Nottingham.*

With 23 Illustrations. 7*s*. 6*d*.

"It is in every way perfectly reliable and in accordance with the most recently acquired knowledge."—*British Medical Journal.*

### Elements of Histology.

By E. KLEIN, M.D., F.R.S,

*Lecturer on General Anatomy and Physiology in the Medical School of St. Bartholomew's Hospital, London.*

NEW AND ENLARGED EDITION. 7*s*. 6*d*.

"A work which must of necessity command a universal success. It is just exactly what has long been a desideratum among students."—*Medical Press and Circular.*

### Surgical Pathology.

By A. J. PEPPER, M.S., M.B., F.R.C.S.,

*Surgeon and Teacher of Practical Surgery at St. Mary's Hospital.*

Illustrated with 99 Engravings.

NEW EDITION, Re-written and Enlarged, 8*s*. 6*d*.

"A student engaged in surgical work will find Mr. Pepper's 'Surgical Pathology' to be an invaluable guide, leading him on to that correct comprehension of the duties of a practical and scientific surgeon which is the groundwork of the highest type of British surgery."—*British Medical Journal.*

CASSELL & COMPANY, LIMITED, *Ludgate Hill, London.*

### Clinical Chemistry.

By CHARLES H. RALFE, M.D., F.R.C.P.,
*Physician at the London Hospital.*

5*s.*

"The volume deals with a subject of great and increasing importance, which does not generally receive so much attention from students as it deserves. The text is concise and lucid, the chemical processes are stated in chemical formulæ, and wherever they could aid the reader suitable illustrations have been introduced."—*The Lancet.*

### Human Physiology.

By HENRY POWER, M.B., F.R.C.S.,
*Late Examiner in Physiology, Royal College of Surgeons of England.*

FOURTH EDITION. REVISED AND ENLARGED. 7*s.* 6*d.*

"The author has brought to the elucidation of his subject the knowledge gained by many years of teaching and examining, and has communicated his thoughts in easy, clear, and forcible language, so that the work is entirely brought within the compass of every student. It supplies a want that has long been felt."—*The Lancet.*

### Materia Medica and Therapeutics.

By J. MITCHELL BRUCE, M.D., F.R.C.P.,
*Lecturer on Materia Medica at Charing Cross Medical School, and Physician to the Hospital.* 7*s.* 6*d.*

*A full account of the many important drugs contained in the Addendum to the British Pharmacopœia, recently issued, will be found in the New Edition.*

"We welcome its appearance with much pleasure, and feel sure that it will be received on all sides with that favour which it richly deserves."—*British Medical Journal.*

### Surgical Applied Anatomy.

By FREDERICK TREVES, F.R.C.S.,
*Surgeon to, and Lecturer on Anatomy at, the London Hospital.*

NEW AND EXTENDED EDITION. 7*s.* 6*d.*

"The author of 'Surgical Applied Anatomy' is an able writer, and is also an authority on purely anatomical questions. There are excellent paragraphs on the anatomy of certain well-known surgical affections, such as hip-joint diseases, constituting a feature quite original in a work of this class, yet in no way beyond its proper scope."—*London Medical Recorder.*

CASSELL & COMPANY, LIMITED, *Ludgate Hill, London.*

### A Manual of Chemistry:

Inorganic and Organic, with an Introduction to the Study of Chemistry. For the Use of Students of Medicine. By ARTHUR P. LUFF, M.D., B.Sc. (Lond.), M.R.C.P.; Fellow of the Institute of Chemistry, &c. &c. With numerous engravings. 7*s.* 6*d.*

"The author is evidently a master of his subject, and the work is one which may be confidently recommended to the student of chemistry."—*Hospital Gazette.*

---

### Surgical Diagnosis:

### A MANUAL FOR THE WARDS.

By A. PEARCE GOULD, M.S., M.B., F.R.C.S.,

*Assistant-Surgeon to Middlesex Hospital.*

7*s.* 6*d.*

"We do not hesitate to say that Mr. Gould's work is unique in its excellence." —*The Lancet.*

---

### Comparative Anatomy and Physiology.

By F. JEFFREY BELL, M.A.,

*Professor of Comparative Anatomy at King's College.*

7*s.* 6*d.*

"The book has evidently been prepared with very great care and accuracy, and is well up-to-date. The woodcuts are abundant and good."—*Athenæum.*

---

### Physiological Physics.

By J. McGREGOR ROBERTSON, M.A., M.B.,

*Muirhead Demonstrator of Physiology, University of Glasgow.*

7*s.* 6*d.*

"Mr. McGregor Robertson has done the student the greatest service in collecting together in a handy volume descriptions of the experiments usually performed and the apparatus concerned in performing them."—*The Lancet.*

CASSELL & COMPANY, LIMITED, *Ludgate Hill, London.*

### CLINICAL MANUALS

FOR

### *Practitioners and Students of Medicine.*

**Complete Monographs on Special Subjects.**

PUBLISHED BY CASSELL & COMPANY.

"A valuable series, which is likely to form, when completed, perhaps the most important Encyclopædia of Medicine and Surgery in the English language."—*British Medical Journal.*

The object of this Series is to present to the Practitioner and Student of Medicine original, concise, and complete monographs on all the principal subjects of Medicine and Surgery, both general and special.

It is hoped that the Series will enable the Practitioner to keep abreast with the rapid advances at present being made in medical knowledge, and that it will supplement for the Student the comparatively scanty information on special subjects contained in the general text-books.

### Diseases of the Ear.

By A. MARMADUKE SHEILD, M.B. Cantab., F.R.C.S. Eng.,

*Assistant-Surgeon and Lecturer on Practical Surgery at St. George's Hospital; late Assistant-Surgeon, Lecturer on Operative Surgery, and Surgeon in Charge of the Aural Department of Charing Cross Hospital.*

With 4 Coloured Plates and 34 Woodcut Illustrations. 10*s.* 6*d.*

### Diseases of the Skin.

An Outline of the Principles and Practice of Dermatology. By MALCOLM MORRIS, F.R.C.S. Ed., Surgeon to the Skin Department, St. Mary's Hospital, London. With 8 Chromo-Lithographs and 17 Woodcuts. *Third Thousand.* 10*s.* 6*d.*

"The most captious critic would find it hard to pick out faults in this most excellent compendium, which worthily sustains the reputation of the series of which it forms a part."—*Edinburgh Medical Journal.*

### On Gall-Stones and their Treatment.

By A. W. MAYO ROBSON, F.R.C.S.,

*Professor of Surgery in the Yorkshire College of the Victoria University; Hon. Surgeon to the General Infirmary at Leeds; Hon. Consulting Surgeon to the Batley Hospital; Late Lecturer on Practical Surgery and Demonstrator of Operative Surgery in the Leeds School of Medicine, &c.*

Illustrated with Twelve Separate Plates. Extra fcap. 8vo, 320 pages, cloth gilt, 9s.

"There can be no question that this book well repays perusal, and will be the work to which all practitioners and students will turn for information on the surgery of the gall bladder."—*Provincial Medical Journal.*

CASSELL & COMPANY, LIMITED, *Ludgate Hill, London.*

### The Pulse.

By Sir W. H. BROADBENT, Bart., M.D., F.R.C.P.,

*Senior Physician to, and Lecturer on Clinical Medicine at, St. Mary's Hospital.*

9*s.*

"There is so much that is interesting and well done that it is hard to emphasise any."—*Hospital.*

### Ophthalmic Surgery.

By R. BRUDENELL CARTER, F.R.C.S.,

*Ophthalmic Surgeon to, and Lecturer on Ophthalmic Surgery at, St. George's Hospital; and*

W. ADAMS FROST, F.R.C.S.,

*Assistant Ophthalmic Surgeon to, and Joint-Lecturer on Ophthalmic Surgery at, St. George's Hospital.*

With Chromo Frontispiece. 9*s.*

"Its clearness and conciseness will cause it to be welcomed by students and young practitioners as an agreeable and useful guide to the modern practice of eye diseases."—*British Medical Journal.*

### Diseases of the Joints.

By HOWARD MARSH, F.R.C.S.,

*Surgeon to, and Lecturer on Surgery at, St. Bartholomew's Hospital, and Surgeon to the Children's Hospital, Great Ormond Street.*

With Chromo Frontispiece. 9*s.*

"This volume is excellently planned. Mr. Marsh brings to bear upon it keen critical acumen."—*Liverpool Medico-Chirurgical Journal.*

### Diseases of the Rectum and Anus.

By CHARLES B. BALL, M.Ch. (Dublin), F.R.C.S.I.,

*Surgeon and Clinical Teacher at Sir P. Dun's Hospital.*

With Chromo Plates. *New Edition.* 9*s.*

"As a full, clear, and trustworthy description of the diseases which it deals with, it is certainly second to none in the language. The author is evidently well read in the literature of the subject, and has nowhere failed to describe what is best up to date. The model of what such a work should be."—*Bristol Medico-Chirurgical Journal.*

### Diseases of the Breast.

By THOMAS BRYANT, F.R.C.S.,

*Surgeon to, and Lecturer on Surgery at, Guy's Hospital.*

With 8 Chromo Plates. 9*s*.

"Mr. Bryant is so well known, both as an author and a surgeon, that we are absolved from the necessity of speaking fully or critically of his work."—*The Lancet.*

### Syphilis.

By JONATHAN HUTCHINSON, F.R.S., F.R.C.S.,

*Consulting Surgeon to the London Hospital and to the Royal London Ophthalmic Hospital.*

*Sixth Thousand.* With 8 Chromo Plates. 9*s*.

"A valuable addition to the series of Clinical Manuals of its publishers, by an expert and accomplished writer, moderate in tone, judicious in spirit, and yet expressing the decided convictions of one whose experience entitles him to speak with authority. The student, no matter what may be his age, will find in this compact treatise a valuable presentation of a vastly important subject. We know of no better or more comprehensive treatise on syphilis."—*Medical News, Philadelphia.*

### Fractures and Dislocations.

By T. PICKERING PICK, F.R.C.S.,

*Surgeon to, and Lecturer on Surgery at, St. George's Hospital.*

9*s*.

"We must express the pleasure with which we have perused the book, and our especial admiration for the lucidity of the author's style, and the simplicity of his directions for the application of apparatus; in the latter respect it is always difficult to combine clearness with brevity, but herein Mr. Pick has been most successful."—*Glasgow Medical Journal.*

### Surgical Diseases of the Kidney.

By HENRY MORRIS, M.B., F.R.C.S.,

*Surgeon to, and Lecturer on Surgery at, Middlesex Hospital.*

With 6 Chromo Plates. 9*s*.

"Mr. Morris writes clearly and forcibly, and handles his subject very thoroughly, so that the reader rises from the perusal of the work impressed with its importance. It would be difficult to find these subjects treated more carefully and thoroughly."—*British Medical Journal.*

### Insanity & Allied Neuroses.

By GEORGE H. SAVAGE, M.D.,

*Late Medical Superintendent and Resident Physician to Bethlem Royal Hospital, and Lecturer on Mental Diseases at Guy's Hospital.*

*Fourth Edition.* 9*s*.

"Dr. Savage's grouping of insanity is practical and convenient, and the observations on each group are acute, extensive, and well arranged."—*The Lancet.*

CASSELL & COMPANY, LIMITED, *Ludgate Hill, London.*

### Intestinal Obstruction.

By FREDERICK TREVES, F.R.C.S.,

*Surgeon to, and Lecturer on Anatomy at, the London Hospital.*

*Third Edition.* 9*s.*

"Throughout the work there is abundant evidence of patient labour, acute observation, and sound reasoning, and we believe Mr. Treves's book will do much to advance our knowledge of a very difficult subject."—*The Lancet.*

### Diseases of the Tongue.

By H. T. BUTLIN, F.R.C.S.,

*Assistant-Surgeon to St. Bartholomew's Hospital.*

With 8 Chromo Plates. 9*s.*

"Mr. Butlin may be congratulated upon having written an excellent manual, scientific in tone, practical in aim, and elegant in literary form. The coloured plates rival, if not excel, some of the most careful specimens of art to be found in the pages of European medical publications."—*British Medical Journal.*

### Surgical Diseases of Children.

By EDMUND OWEN, M.B., F.R.C.S.,

*Senior Surgeon to the Children's Hospital, Great Ormond Street, and Surgeon to, and Co-Lecturer on Surgery at, St. Mary's Hospital.*

*Second Edition.* With 4 Chromo Plates. 9*s.*

"Mr. Owen's volume will rank as an invaluable *résumé* of the subject on which it treats, and should readily take its place as a reliable and compact guide to the surgery of children."—*Medical Press and Circular.*

### Food in Health and Disease.

By I. BURNEY YEO, M.D., F.R.C.P.,

*Physician to King's College Hospital, and Professor of Clinical Therapeutics, King's College.*

*Fifth Thousand.* 9*s.*

"We think that Dr. Yeo is to be congratulated on having accomplished his desire; we became more and more favourably impressed with the work as we went through the various chapters, and we have no doubt that it will attain, as it deserves, a great success."—*The Lancet.*

*Published by Cassell & Company.*

"This Manual of Surgery is unique of its kind."

—*Medical Press and Circular.*

*Complete in Three Volumes, each containing about* 600 *pages, fcap.* 8vo, *fully Illustrated.* 7*s.* 6*d. each.*

### A Manual of Surgery.

### In Treatises by various Authors.

EDITED BY

**FREDERICK TREVES, F.R.C.S.,**

*Surgeon to, and Lecturer on Anatomy at, the London Hospital Hunterian Professor at the Royal College of Surgeons of England;*

AND

**Containing Contributions by leading Physicians and Surgeons.**

*Seventh Thousand.*

"It would be almost impossible to find at present any work in which the subjects treated of are written more clearly or concisely. The editor has had a difficult task to accomplish in the production of this work, and we congratulate him on the successful result."—*The Lancet.*

"It is undoubtedly one of the most compendious surgical works, and from the variety of its authorship may be considered somewhat representative of the surgical opinion of these islands. The illustrations are excellent."—*Liverpool Medico-Chirurgical Journal.*

### A Manual of Medical Treatment or Clinical Therapeutics.

By I. BURNEY YEO, M.D., F.R.C.P.

With Illustrations. *Fourth Edition.* Two Vols., 21s.

"Dr. Yeo has dealt with the treatment of disease not as is ordinarily the case, in the form of an appendix to a disquisition on the sources and physiological actions of remedies, but in its more rational and instructive place—namely, after an exposition of the diseases themselves. We may well congratulate the author on having written a book which deserves, and will doubtless command, a wide circle of readers, who will appreciate its value and utility."—*The Lancet.*

"It will be seen that the term medical treatment is here used in its widest sense, that it not only includes the indications for and administration of drugs, but embraces questions of diet, hygiene, hydrotherapy, electrical and climatic treatment, as well as the prevention of the spread of disease to others. This work, with its information always well up to date, may be warmly recommended, as its use will help to disseminate a knowledge of rational therapeutics in the strictest meaning of the term."
—*British Medical Journal.*

CASSELL & COMPANY, LIMITED, *Ludgate Hill, London.*

**Issued Yearly. About 500 pages, 8vo, *price* 7s. 6d.**

### The Year-Book of Treatment:

*A Critical Review for Practitioners of Medicine.*

"In this useful publication there is no indication of any decline in the industry of the contributors, in the collection of fit materials, or in the critical value of their remarks on new therapeutic procedures. On the contrary, experience seems to have perfected their judgment. In fact, the whole volume is full of good things, and will prove a great boon to the busy practitioner. . . . It is *a book of extreme value* to all who in these busy times find it difficult to keep pace with the ever-advancing march of the science and art of medicine."—*The Lancet.*

"This handbook contains *a wonderfully complete summary-review of the methods of treatment*, new or resuscitated, which have been advocated during the year with which it deals."—*British Medical Journal.*

"The cause of the signal favour with which the work has been welcomed is probably to be found in the long list of well-known names of contributors to the work, each division being written by a different hand."—*Medical Press and Circular.*

"A vast mass of valuable information and accumulated experience is published each week, but scattered in the numerous periodicals in English, French, and German; and in the midst of much that would benefit no one to remember, it is very apt to be overlooked. It is obvious that the busy practitioner cannot wade through this heterogeneous collection, and he will hail with delight a guide which gives him a selection of the new methods of treatment in use both here and on the Continent, and at the same time an estimate of their value by men who, by their special reading and hospital experience, have made themselves pre-eminent in the branch they have supervised. . . . *We can strongly recommend the book to all practitioners*, and we shall not be exaggerating if we say that *this book is one of that class which it is an economy to possess.*"—*London Medical Recorder.*

"This valuable annual may be commended to the attention of those who are on the outlook for *a comprehensive and reliable summary of therapeutic progress in every branch of medicine and surgery.*"—*Glasgow Medical Journal.*

"That *this book has met a long-felt want* its rapid and extensive sale abundantly testifies."—*Liverpool Medico-Chirurgical Journal.*

CASSELL & COMPANY, LIMITED, *Ludgate Hill, London.*

### A Handbook of Nursing for the Home and for the Hospital.

By CATHERINE J. WOOD, Lady Superintendent of the Hospital for Sick Children, Great Ormond Street. TENTH AND CHEAP EDITION. Price 1*s.* 6*d.*; cloth, 2*s.*

"A book which every mother of a family ought to have, as well as every nurse under training."—*Guardian.*

"The best book of its kind."—*Independent.*

---

### A Handbook for the Nursing of Sick Children.

With a few Hints on their Management. By CATHERINE J. WOOD. 2*s.* 6*d.*

"Miss Wood's book is succinct, clearly written, and goes straight to the heart of each detail in a thoroughly business-like fashion."—*Health.*

---

*NEW AND REVISED EDITION.*

### Medical Handbook of Life Assurance.

For the use of Medical and other Officers of Companies. By JAMES EDWARD POLLOCK, M.D., F.R.C.P.; and JAMES CHISHOLM (Fellow of the Institute of Actuaries, London). 7*s.* 6*d.*

---

### Vaccination Vindicated:

Being an Answer to the Leading Anti-Vaccinators. By JOHN C. MCVAIL, M.D., D.P.H.Camb. 5*s.*

---

### The Natural History of Cow-Pox and Vaccinal Syphilis.

By CHARLES CREIGHTON, M.D. 3*s.*

CASSELL & COMPANY, LIMITED, *Ludgate Hill, London.*

### Diet and Cookery for Common Ailments.

By A FELLOW OF THE ROYAL COLLEGE OF PHYSICIANS and PHYLLIS BROWNE. 5*s*.

---

### Advice to Women on the Care of their Health,

Before, During, and After Confinement. By FLORENCE STACPOOLE, Diplomée of the London Obstetrical Society, &c. &c. Paper covers, 1*s*.; or cloth, 1*s*. 6*d*.

"The writer of the present volume has shown much tact in her way of setting forth those simple rules which all may understand and practise, and of avoiding those points which would only mystify and injure. . . . Well and clearly written and full of practical hints."—*British Medical Journal*.

---

### Our Sick, and How to Take Care of Them:

or, Plain Teaching on Sick Nursing at Home. By FLORENCE STACPOOLE. Paper covers, 1*s*.; or cloth, 1*s*. 6*d*.

"'Our Sick' is a valuable handbook for home nursing in rich or poor circumstances, the latter being especially studied, and no useful hint left out."—*Guardian*.

---

### The Treatment of Typhoid Fever,

Especially by "Antiseptic" Remedies. By I. BURNEY YEO, M.D., F.R.C.P. 1*s*. 6*d*.

---

### An Address in School Hygiene.

By CLEMENT DUKES, M.D. Lond., M.R.C.P. Lond. Demy 8vo. 1*s*.

*Authoritative Work on Health by Eminent Physicians and Surgeons.*

### The Book of Health.

A Systematic Treatise for the Professional and General Reader upon the Science and the Preservation of Health, **21s.**; roxburgh, **25s.**

CONTENTS.

By SIR W. S. SAVORY, BART., F.R.S.—INTRODUCTORY.

By SIR RISDON BENNETT, M.D., F.R.S.—FOOD AND ITS USE IN HEALTH.

By T. LAUDER BRUNTON, M.D., F.R.S.—THE INFLUENCE OF STIMULANTS AND NARCOTICS ON HEALTH.

By SIR J. CRICHTON-BROWNE, LL.D., M.D.—EDUCATION AND THE NERVOUS SYSTEM.

By JAMES CANTLIE, F.R.C.S.—THE INFLUENCE OF EXERCISE ON HEALTH.

By FREDERICK TREVES, F.R.C.S.—THE INFLUENCE OF DRESS ON HEALTH.

By J. E. POLLOCK, M.D.—THE INFLUENCE OF OUR SURROUNDINGS ON HEALTH.

By J. RUSSELL REYNOLDS, M.D., F.R.S.—THE INFLUENCE OF TRAVELLING ON HEALTH.

By SHIRLEY MURPHY, M.R.C.S.—HEALTH AT HOME.

By W. B. CHEADLE, M.D.—HEALTH IN INFANCY AND CHILDHOOD.

By CLEMENT DUKES, M.D.—HEALTH AT SCHOOL.

By HENRY POWER, F.R.C.S.—THE EYE AND SIGHT.

By G. P. FIELD, M.R.C.S.—THE EAR AND HEARING.

By J. S. BRISTOWE, M.D., F.R.S.—THE THROAT AND VOICE.

By CHARLES S. TOMES, F.R.S.—THE TEETH.

By MALCOLM MORRIS.—THE SKIN AND HAIR.

By SIR JOSEPH FAYRER, K.C.S.I., F.R.S., and J. EWART, M.D.—HEALTH IN INDIA.

By HERMANN WEBER, M.D.—CLIMATE AND HEALTH RESORTS.

Edited by MALCOLM MORRIS, F.R.C.S. Ed.

"'The Book of Health,'" says the *Lancet*, "is what it aims to be—authoritative, and must become *a standard work of reference* not only with those who are responsible for the health of schools, workshops, and other establishments where there is a large concourse of individuals, but to *every member of the community* who is anxious to secure the highest possible degree of healthy living for himself and for his family."

---

CASSELL & COMPANY'S COMPLETE CATALOGUE, *containing particulars of upwards of One Thousand Volumes, including Bibles and Religious Works, Illustrated and Fine-Art Volumes, Children's Books, Dictionaries, Educational Works, History, Natural History, Household and Domestic Treatises, Science, Travels, &c., together with a Synopsis of their numerous Illustrated Serial Publications, sent post free on application.*

CASSELL & COMPANY, LIMITED, *Ludgate Hill, London; Paris & Melbourne.* [2.95.

